# Waveguide-Integrated Colloidal Nanocrystal Supraparticle Lasers

**DOI:** 10.1021/acsaom.3c00312

**Published:** 2023-11-15

**Authors:** Pedro Urbano Alves, Benoit J. E. Guilhabert, John R. McPhillimy, Dimitars Jevtics, Michael J. Strain, Matěj Hejda, Douglas Cameron, Paul R. Edwards, Robert W. Martin, Martin D. Dawson, Nicolas Laurand

**Affiliations:** † Institute of Photonics, Department of Physics, SUPA, Technology and Innovation Centre, University of Strathclyde, 99 George Street, Glasgow G1 1RD, U.K.; ‡ Department of Physics, SUPA, University of Strathclyde, John Anderson Building, 107 Rottenrow, Glasgow G4 0NG, U.K.

**Keywords:** semiconductor nanocrystals, microresonators, whispering gallery modes, self-assembly, supraparticles, transfer printing, integrated photonics

## Abstract

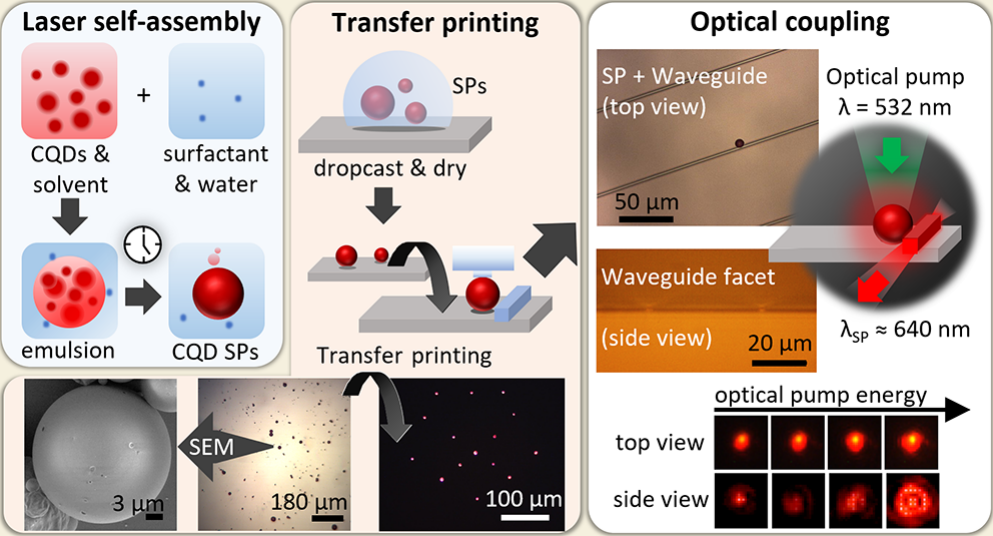

Supraparticle (SP) microlasers fabricated by the self-assembly of colloidal nanocrystals have great potential as coherent optical sources for integrated photonics. However, their deterministic placement for integration with other photonic elements remains an unsolved challenge. In this work, we demonstrate the manipulation and printing of individual SP microlasers, laying the foundation for their use in more complex photonic integrated circuits. We fabricate CdS_x_Se_1−x_/ZnS colloidal quantum dot (CQD) SPs with diameters from 4 to 20 μm and Q-factors of approximately 300 *via* an oil-in-water self-assembly process. Under a subnanosecond-pulse optical excitation at 532 nm, the laser threshold is reached at an average number of excitons per CQD of 2.6, with modes oscillating between 625 and 655 nm. Microtransfer printing is used to pick up individual CQD SPs from an initial substrate and move them to a different one without affecting their capability for lasing. As a proof of concept, a CQD SP is printed on the side of an SU-8 waveguide, and its modes are successfully coupled to the waveguide.

## Introduction

Colloidal semiconductor nanocrystals (NCs) are known for their size-tunable electronic and optical properties, discrete density of states, and low-temperature solution processing,^1−4^ which make them very attractive as the gain medium of lasers.^5^ Additionally, NCs can be used on many different material platforms and have great prospects for integrated photonics where they could form the basis of miniature optical sources and nonlinear elements.^6^ In this context, if their desired performance and scalability can be achieved, then they have the potential to enable photonic chips of future generations.

Several different NC laser geometries have been reported to date, *e.g.*, Fabry–Pérot cavities,^7^ microring resonators,^8^ vertical cavities implemented with distributed Bragg reflectors,^9^ distributed feedback cavities,^10^ and microsphere cavities where dielectric spheres are doped or coated with NCs.^11,12^ The fabrication of such lasers typically requires top-down patterning of the nanocrystals at a submicron level (*e.g.*, using photo or contact lithography) or a way to add them to an optical microcavity that is fabricated separately. An elegant fabrication alternative has been recently reported, where the NCs self-assemble from the bottom up in solution to form both the gain material and the laser cavity. This approach leads to supraparticles (SPs), often in the form of microspheres, with a crystalline structure.^13,14^ Such supraparticles take advantage of the high refractive index of the densely packed semiconductor nanocrystals and the shape of the self-assembled structure to efficiently trap light, thus generating a whispering gallery mode (WGM) cavity.^15^ In contrast to lasers that integrate NCs by coating or doping resonators made of another material, SPs do not require a separate cavity, thereby simplifying the fabrication process.^14^ The high density of NCs in SPs also enhances the resonances, which can result in an increase of absorption efficiency by more than 2 orders of magnitude when compared to dispersed NCs .^15^ This enhancement is a prerequisite to achieving efficient micron-scale lasers.

An early report of SP lasers made with CdSe/CdS colloidal quantum dots (CQDs), a subclass of NCs, has shown WGMs with quality factors (*Q*-factors) of up to 320.^13^ The laser threshold fluence of these CdSe/CdS microspheres was approximately 100 μJ/cm^2^ for a 100–200 fs pulse pumping (repetition rate of 1 kHz and spot size 10 μm full width at half-maximum) and the emission exhibited a predominantly linear polarization.^13^ Enhanced excitonic coupling has also been observed in CQD-based SPs, which is promoted by the degree of order and distance between CQDs in the structure.^15^ In other works, it has been shown that the NC building blocks can be chosen for SP laser oscillation at a desired wavelength or at several wavelengths simultaneously by assembling a blend of alloyed CdSSe/ZnS CQDs having different characteristics.^16,17^ The building blocks can also be made of other NCs, such as colloidal quantum wells (CQWs). In this particular case, SPs made of CdSe CQWs were reported to have a lower lasing threshold and a higher quantum yield than CdSe CQDs.^18^ Single-mode laser emission has also been reported for 1.5–5 μm SPs made of CdSe/ZnS core–shell CQDs, which were tested for *in vitro* and *in vivo* biological imaging. The inter-CQD distance in these SPs was shortened *via* ligand exchange in order to further increase the density of CQDs and in turn the optical gain.^19^

SPs represent a novel family of NC lasers that retain the attractive properties of NCs, while also offering the advantage of combining a microsize resonating structure and straightforward fabrication. SP lasers are being researched as unbound structures in biological and medical applications,^19^ but interest in their use when embedded within optoelectronics is now accelerating.^20^ Nevertheless, there is a lack of techniques capable of manipulating individual SPs for their deterministic placement on a substrate or within a system, which is a current major obstacle to their implementation in integrated photonics.

In this work, microtransfer printing is proposed and demonstrated as a solution to the challenge described above. First, SPs made of CdS_*x*_Se_1–*x*_/ZnS CQDs ranging approximately between 4 and 20 μm in diameter are fabricated and characterized individually under optical pumping. The origin of the whispering gallery mode lasing in these SPs is confirmed by comparing numerical simulations of the modes with optical pumping experiments as well as through cathodoluminescence measurements. The SP emission intensity below the threshold is fitted and studied using a modified Poissonian function model in order to extract ⟨*N*⟩, the average number of excitons per CQD in the microlaser, at different excitation levels and for different sizes of lasers. ⟨*N*⟩ is a parameter that relates to the population inversion within the SP, and its value at the threshold is a performance benchmark for such microlasers. Microtransfer printing^21−23^ is then shown to be a viable method to accurately select and transport individual SP lasers between substrates without damaging them. As proof of concept, an SP is transfer-printed next to a waveguide and optically pumped. Specific laser modes of the SP are successfully coupled and detected at the end of the waveguide (output facet). This successful demonstration is a pioneering step toward the integration of these microlasers into more complex optoelectronics applications.

## Results and Discussion

### Synthesis of SPs

**Figure 1:**
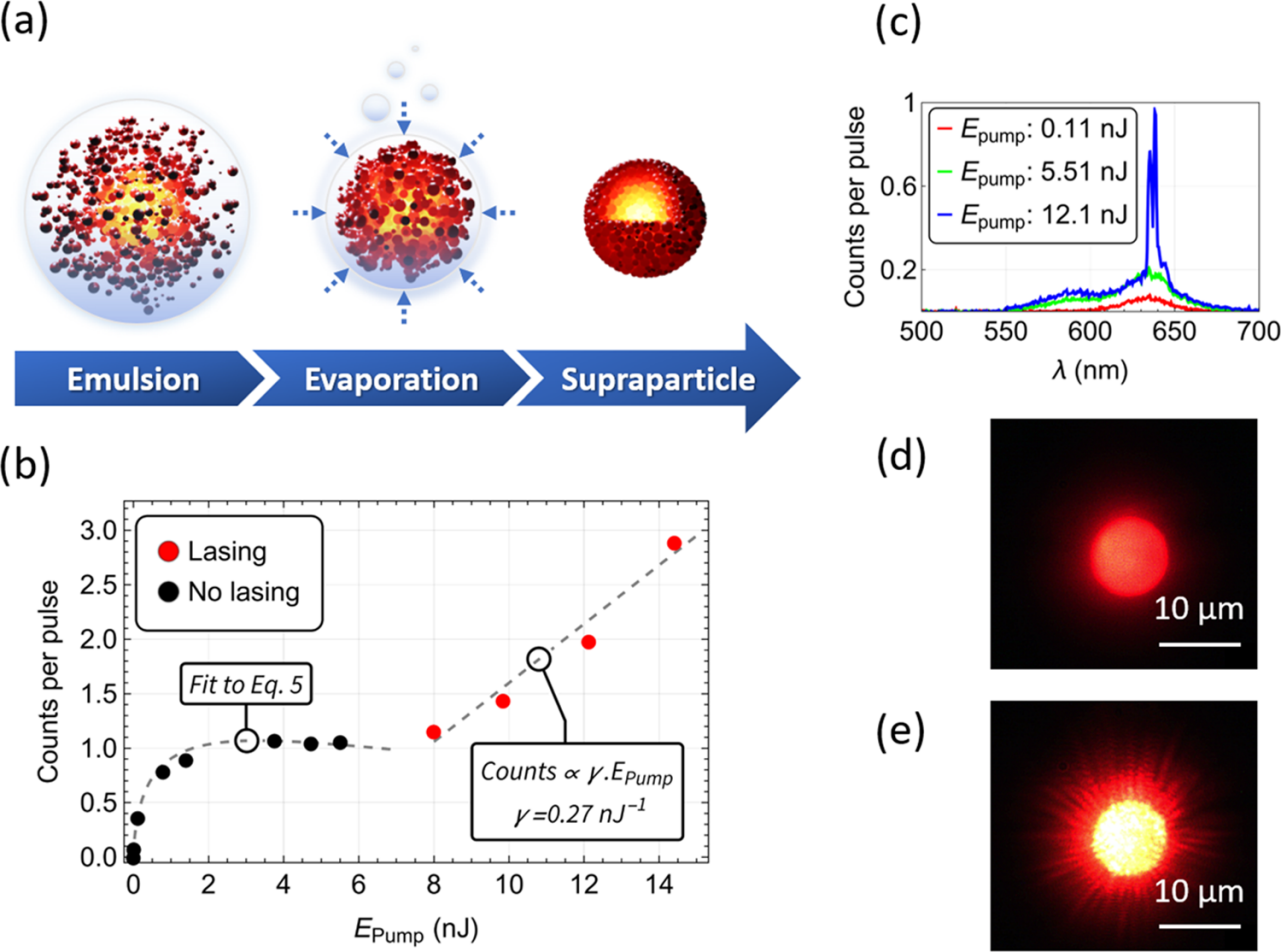
Illustration of the nucleation process occurring inside the emulsion droplets that leads to SPs (a); emission intensity versus pump energy, with laser threshold at approximately 7 nJ, (b) and emission spectra (c) of an SP with a diameter of 9.8 ± 0.5 μm; micrographs of an SP under optical pumping (λ_pump_ = 532 nm; see the Optical Characterization Section) below and above the lasing threshold (d, e). The full optical setup can be seen in Figure S3.

SPs were synthesized from CdS_*x*_Se_1–*x*_/ZnS CQDs with a nominal size of 6.0 ± 0.5 nm and an intrinsic emission peak of 630 nm (Quantum Dots Section). The synthesis followed an oil-in-water self-assembly process and used poly(vinyl alcohol) (PVA) as the surfactant (emulsifier) to stabilize the emulsion (see the Synthesis and Characterization of the SPs section). Water-based solutions are polar and therefore immiscible in nonpolar organic solvents (*e.g.*, chloroform). PVA adsorbs at the interface between these two phases, decreasing the surface tension and promoting stable emulsions. This synthesis used CQDs in chloroform and PVA in Milli-Q water as the oil and water phases, respectively. After mixing the two phases and while the chloroform evaporates, the building blocks (CQDs) inside each emulsion droplet begin to nucleate and grow into SPs of tightly packed CQDs^13,14^ (Figure 1a). The average size of the emulsion droplets formed upon mixing these two phases is mainly determined by the volume of the oil phase and the concentration of the surfactant in the water phase. Likewise, the size of the self-assembled SP is determined by the amount of CQDs inside the emulsion droplet, which depends on the initial concentration of CQDs in the oil phase and on the volume of the emulsion droplet. The self-assembly process finishes once all of the chloroform inside the emulsion droplets is evaporated. A final washing step was used in this procedure to remove traces of PVA from the surfaces of SPs. The SPs formed this way were on average 2.8 ± 1.7 μm in radius (Figure S2).

### Characterization of SPs

Sixteen SPs of different sizes were drop-casted on silica and characterized individually with a custom-made microphotoluminescence (μPL) setup (Figure S3). The optical pump source was a 0.76 ns pulse width microchip laser (λ = 532 nm) at a repetition rate of 7.1 kHz, and the beam spot area at the sample was 2.88 × 10^–7^ cm^2^. The energy of the optical pump was controlled and measured with an attenuator wheel and a power meter, respectively (see the Optical Characterization Section).

The laser transfer function (emission intensity versus pump intensity) of a 9.8 ± 0.5 μm diameter SP can be seen as a typical example in Figure 1b. The lasing threshold is defined as the value of pump energy above which there is a drastic change in the slope of intensity (Figure 1b) and narrow emission peaks dominate. This SP occurs at an incident pump energy of 7 nJ. Above 7 nJ, spectrally narrow laser modes develop in the 635–640 nm wavelength range on top of the broader spontaneous emission pedestal (Figure 1c). The main photoluminescence (PL) peak, around 630 nm, corresponds to the excitonic and biexcitonic transitions of the CQDs, which is predominant at low pump energies (Figure 1c, red spectrum).^2^ At higher pump energies, emission from the negatively charged biexcitons or triexcitons and other multiexcitons of higher order can also be detected at around 580 nm (Figure 1c, green and blue spectra).^2,24,25^ In addition to the clear threshold behavior seen in the emission intensity (Figure 1b), and the change in the emission spectrum (Figure 1c), the differences between SPs below and above the lasing regime are also observed in the microscope images (Figure 1d,1e, respectively), with an evident transition in intensity and the appearance of a WGM lasing pattern characterized by the deep red corona on the SP periphery. The wavelengths of the lasing peaks observed experimentally in Figure 1c also match with the resonant wavelengths for the transverse electric and magnetic modes calculated numerically using the modal equations (Figure S4 and Table S1). The measurements on the resonance frequencies of a microsphere and the analysis of an analogous CQD SP below and above the laser threshold indicate that the laser emission arises from WGM (Figure S4 and Table S1). These results are consistent with previous reports in the literature.^13,15^

Laser emission results described in Figure 1c are typical of the lasing SPs characterized in this work. In general, the different SPs display WGM laser oscillation with spectrometer-resolution-limited peaks between 625 and 655 nm. They oscillate on one or several angular modes, depending on the SP size and pumping levels (Table S2).

**Figure 2:**
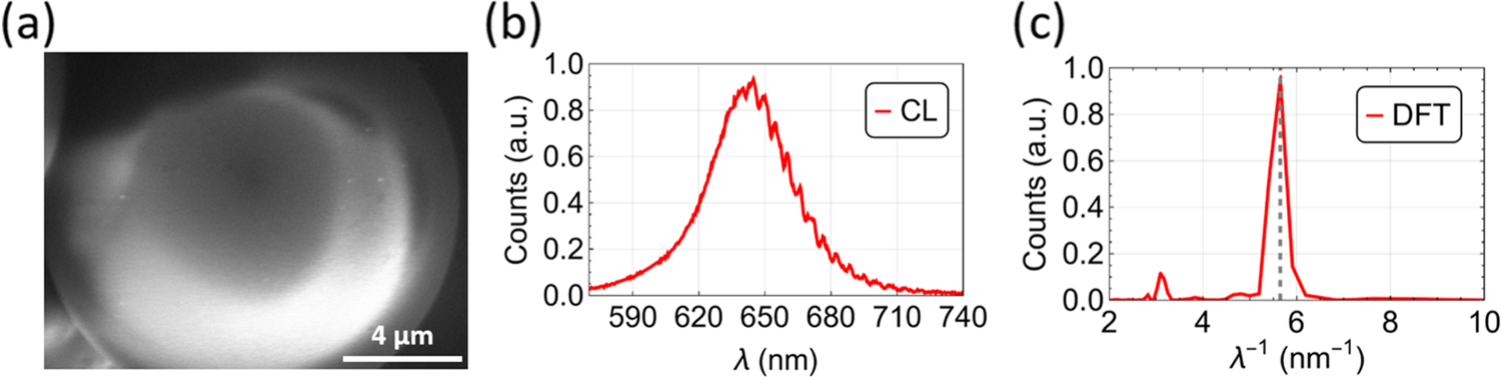
SEM image of an SP of approximately 14 μm in diameter (a) and its CL spectrum (b) and discrete Fourier transform analysis (c). The *Q-*factor estimated from the WGMs was 295 ± 15.

The WGMs of a typical SP were also observed below the threshold using the scanning electron microscopy (SEM) technique of cathodoluminescence (CL) while imaging the SP at the same time (Figure 2a). The CL originates from the electron-beam excitation of the SP, which leads to the subsequent emission of photons. The CL spectrum was acquired with a spectrometer coupled to the SEM microscope (Figure 2b). Observing WGMs below the threshold proved easier with CL than with the PL setup because of a higher contrast between the WGM signature and the background luminescence. The WGMs are evidenced as the spectral modulation that is seen at the long-wavelength side of the CL spectrum (Figure 2b); the modes are not visible at lower wavelengths because of self-absorption (see the overlap between the emission and absorption spectra of CQDs in Figure S1). The pseudofree spectral range (pseudo-FSR), *i.e.*, the frequency separation between WGMs of consecutive angular modes, is then obtained using a discrete Fourier transform of the CL spectrum (Figure 2c). The pseudo-FSR of spherical microresonators, Δν_*n*,*l*_^Δ*l*^, is correlated to the radius of the sphere, *r*, as follows: , where the indices *n* and *l* correspond to the order of the spherical harmonic that describes the radial and angular field distributions, respectively, *c* is the speed of light in vacuum, and *N* is the refractive index.^26^ From the SEM image, the diameter of the SP in Figure 2a is 14.0 ± 0.5 μm. This value is consistent with the 13.7 ± 0.5 μm calculated using the pseudo-FSR correlation for consecutive modes^26^ and a refractive index of *N* = 1.7, which is the expected refractive index value of a Cd-based CQD medium.^27^ The *Q*-factor of the modes can be calculated from Figure 2b as *Q* = λ/Δλ, where λ corresponds to the wavelength of the mode propagated in the cavity and Δλ to the full width at half-maximum of that mode. Here, the *Q*-factor is estimated to be 295 ± 15, which is consistent with the *Q*-factors previously reported on SPs of approximately the same size and composition self-assembled *via* microfluidics.^13^

From the SEM characterization in this work and morphology reports in the literature, these CQD SPs are also expected to have a partially crystalline structure.^13,14^

### Average Number of Excitons per CQDs at Threshold: Modeling the Spontaneous Emission of SPs

The sublinear evolution of the emission intensity of SPs versus pump energy below the laser threshold (Figure 1b) indicates that lasing oscillation is reached in a regime where there is more than one exciton per CQD on average. Accurate estimations of the average number of excitons can be performed either *via* numerical gain modeling or transient absorption measurements.^13,28^ However, these require heavy computation or complex setups to perform the measurements. Here, the average number of excitons in SPs is estimated by establishing a parallelism to CQDs.

The spontaneous emission, *I*_QD_ (*k*,⟨*N*⟩), of a CQD is proportional to the Poisson distribution^7,29,30^
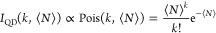
1where ⟨*N*⟩ is the average number of excitons in the CQD during the acquisition. The transition from a given excitonic state identified by *k* () has its own signature emission wavelength, and the emission probability from this *k*th-state, Pois(*k*,⟨*N*⟩), can be estimated provided that the average number of excitons ⟨*N*⟩ in the CQD is known. The average number of excitons ⟨*N*⟩ can be accurately described provided that the time-integrated intensities of the incident (*I*_*i*_), transmitted (*I*_*t*_), and specularly reflected (*I*_*r*_) pump beams are known (*e.g.*, by measuring them in an integrating sphere) or it can be approximated to a power law as a function of the incident energy.^29,30^

In the case of SPs, ⟨*N*⟩ is expressed as in eq 2
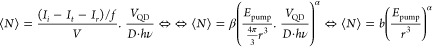
2where *f* corresponds to the frequency of the pump laser,  corresponds to the pumped volume (assumed for simplicity to be the volume of the whole SP), *D* is the density fraction of CQDs, *V*_QD_ is the mean volume of a single CQD, *E*_pump_ is the pump energy, *hν* is the pump photon energy, α and β are the power law constants, and *r* is the radius of the SP.

While the Poisson distribution is suitable to describe the emission of single CQDs or of an ensemble of noninteracting CQDs,^31^ it is less evident in the case of SPs as these are made of many densely packed CQDs, each with their own ⟨*N*⟩. Energy transfer between CQDs is prone to occur in densely packed CQDs, and therefore their emission cannot be considered fully independent. Furthermore, even if the approximation of noninteracting CQDs was valid, it is not always possible to discriminate between all of the different excitonic transitions in the emission spectrum of such ensembles as CQDs since small changes in size will lead to the same excitonic state emitting at slightly different wavelengths. In addition to that, different excitonic states *k*_*i*_,  can also recombine and emit at similar wavelengths, which makes it impossible to describe the emission intensity as a single discrete Pois(*k*,⟨*N*⟩). A sum of discrete Poisson distributions, each describing the emission of a CQD in the SP, would also lead to a very large number of fitting parameters. In order to estimate ⟨*N*⟩ in SPs, a modified model based on the continuous analogue of the Poisson distribution is therefore proposed and applied below.

An average exciton state *k̅* () is defined to describe the combined emission probability distribution of multiple states. The population of excitons and biexcitons, with *k* = 1 and *k* = 2, respectively, has an emission peak that sits at approximately 630 nm and is assigned as *k̅*_1_ (*k̅*_1_ ≤ 2). The emission probability distribution for the population of the higher multiexcitonic states (*k* = 3, 4, 5···) is assigned as *k̅*_2_ (*k̅*_2_ > 2) and refers to the first multiexcitons with emission at 580 nm, approximately.

The continuous Poisson distribution, Pois_*C*_ (*k̅*, ⟨*N*⟩), is defined as^32^
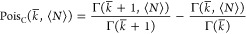
3where Γ(*k̅*,⟨*N*⟩) is the incomplete γ function and Γ(*k̅*) is the Euler γ function. The emission of SPs is then proportional to their volumes and emission probability distribution at those given wavelengths
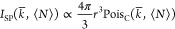
4

A multi-non-linear model fit^33^ is performed on the emission intensity of the two emission wavelengths centered at 630 and 580 nm (Data_630nm_ and Data_580nm_) to find the best fitting parameters and uncertainties for *a*, *b*, α, *k̅*_1_, and *k̅*_2_
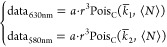
5

**Figure 3:**
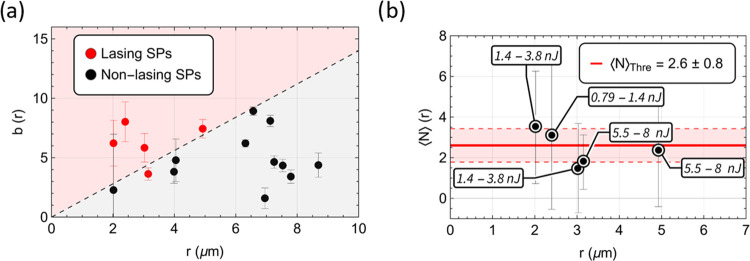
Study on the free parameter b (from Table S3) as a function of the SP radius (a), extracted using eqs 3–5. The black data points correspond to the SPs that did not achieve lasing, and the red data points to those that did. The two data sets are visibly separable (dashed line), suggesting that the capability of an SP of a given size to operate as a laser is strongly intertwined with the parameter b. The average number of excitons ⟨*N*⟩ at a laser threshold was then calculated for the lasing SPs (b) based on the fitting parameters (eq 2 and Table S3). The optical pump energies required to reach the laser threshold were extracted from Table S2 and included in the callouts. SPs were optically pumped at λ_pump_ = 532 nm (see the Optical Characterization Section). The full optical setup can be seen in Figure S3.

Sixteen randomly chosen SPs with sizes ranging between approximately 4 and 18 μm in diameter had their emission intensity recorded at different *E*_pump_ below the laser threshold (Figure S5 and Table S3). The set of parameters (*a*, *b*, α, *k̅*_1_, and *k̅*_2_) is then estimated for each SP by fitting the experimental data intensity peaks at around 630 and 580 nm to eq 5. The data from the 16 sets are split according to each parameter and analyzed as a function of the size of SPs (Figures 3 and S6).

Figure 3a shows that the parameter *b* ranges between 0 and 10 and is independent of the radius, *r*, of the SP. This random distribution of *b* and its independence on the size of the SP is ascribed to fluctuations in the density of CQDs, *D*, between SPs, as well as other factors (*e.g.*, density of excess ligands in the SP, remnants of surfactant or debris in the SP, defects in the composition of SPs) affecting the pump absorption and collection of the emission. In this figure, the SPs that reached the laser threshold are identified by red data points. The relationship between *b* and *D* (eq 2) and the trend of the interface between lasing SPs and nonlasing SPs marked by the dashed line confirms that the density of CQDs plays a role in the laser threshold energy. For the current setup, the density of CQDs would actually need to decrease for the SPs of large diameter to reach a threshold. This appears counterintuitive but could in fact be explained by the reduced coverage of pump light in larger SPs due to the fixed size of the beam spot. This causes larger SPs to have inhomogeneously excited CQDs, thus favoring reabsorption over emission in their WGMs.

The parameter α stays approximately the same regardless of the size of the SP (α = 0.35 ± 0.06; Figure S6), and the fact that it is lower than 1 indicates the existence of nonlinear nonradiative processes during the recombination of electron–hole pairs.

Some fluctuations attributed to the overlap between the optical pump spot size and the SP can be seen for parameter *a* (*a* = 0.06 ± 0.03; Figure S6). The overlap affects the pump light coupled into the SP and therefore the counts detected on the spectrometer.

The two average exciton states *k̅*, corresponding to the exciton/biexciton (*k̅*_1_) and multiexciton (*k̅*_2_) populations in the SP, show that the exciton/biexciton states plateau at *k̅*_1_ = 1.6 ± 0.4, and the multiexciton states plateau at *k̅*_2_ = 3.2 ± 0.6 (Figure S6).

Five out of the eight SPs of radii between 2 and 5 μm reached a threshold. The laser threshold was reached for an average number of excitons of approximately ⟨*N*⟩ = 2.6 ± 0.8 (Figure 3b). This result is consistent with the average number of excitons per dot of ⟨*N*⟩ = 2.5 modeled numerically in the state-of-the-art SPs made of type I CQDs.^13^

### Microtransfer Printing and Waveguide Integration of SP Lasers

**Figure 4:**
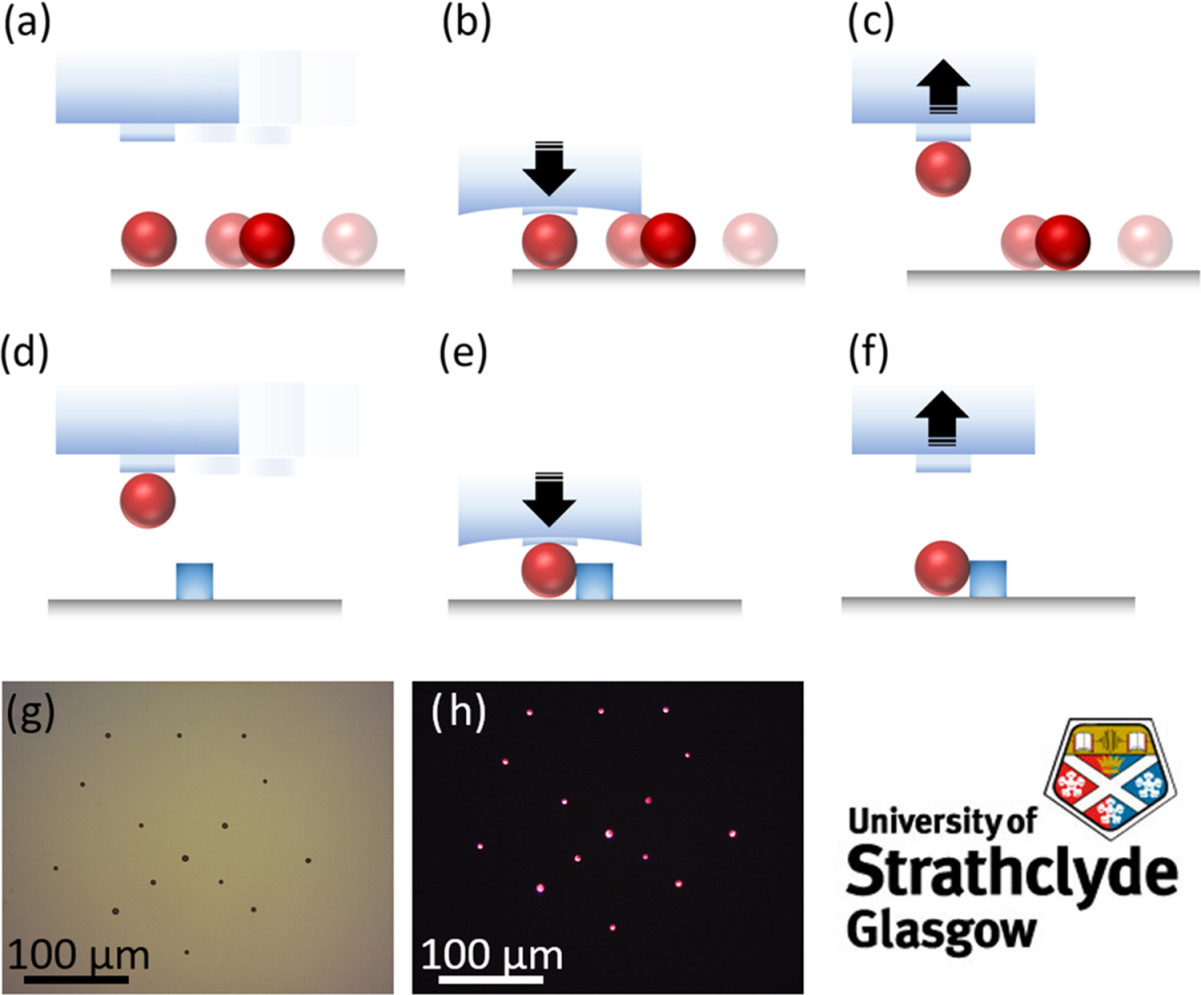
Illustration of the transfer printing process applied to the waveguide coupling of an SP: selection of the SP (a); pick up (b, c); selection of the target destination, *e.g.*, substrate with a waveguide (d); and drop off (e, f). Proof of concept with 15 SPs transfer-printed onto a PDMS substrate to mimic the University of Strathclyde logo under white light (g) and a UV light lamp, λ_lamp_ = 365 nm (h). Logo used with permission from University of Strathclyde, Glasgow.

Manipulation of SPs between substrates (bare glass to bare poly(dimethylsiloxane), *i.e.* PDMS, and bare glass to glass with polymeric waveguides) was achieved *via* transfer printing, a technique capable of combining hard materials such as epitaxial semiconductor structures with dissimilar materials that otherwise would not be compatible.^21−23,34^ This technique has been demonstrated to print thin LEDs onto diamond and silica with submicron resolution,^22^ epitaxial nanowires onto polymers,^34^ and more recently on the deterministic integration of nanowires, dense integration of micron devices, and advanced transfer printing methods.^35−37^ Prior to this work, however, the technique had not been explored for self-assembled microcavities made from colloidal materials. The transfer printing setup in this study used a modified dip-pen nanolithography system with a transparent polymer stamp made of PDMS and an in-line camera that allows visualization of the samples through the stamp. A schematic of the process is shown in Figure 4a–f. The stamp is brought into contact with a single SP sitting on a donor substrate (*e.g.*, glass slide with SPs drop-cast on it), and when the stamp is peeled from the donor substrate, the adhesion is strong enough to lift the SP from the donor onto the surface of the PDMS stamp. Likewise, when the stamp is brought into contact with the receiving substrate and then retracted, the SP adheres to the receiving substrate. SPs can then be individually selected with the stamp, picked up, moved, and dropped off at a desired location. The PDMS stamp (length × width: 100 μm × 200 μm) used in the transfer printing process was cast from a mold using a silicon elastomer and curing agent at a ratio of 10:1. The tip in the center, used to pick up and drop off SPs, corresponds to a small extrusion of the main block of PDMS (length × width: 10 μm × 30 μm).

**Figure 5:**
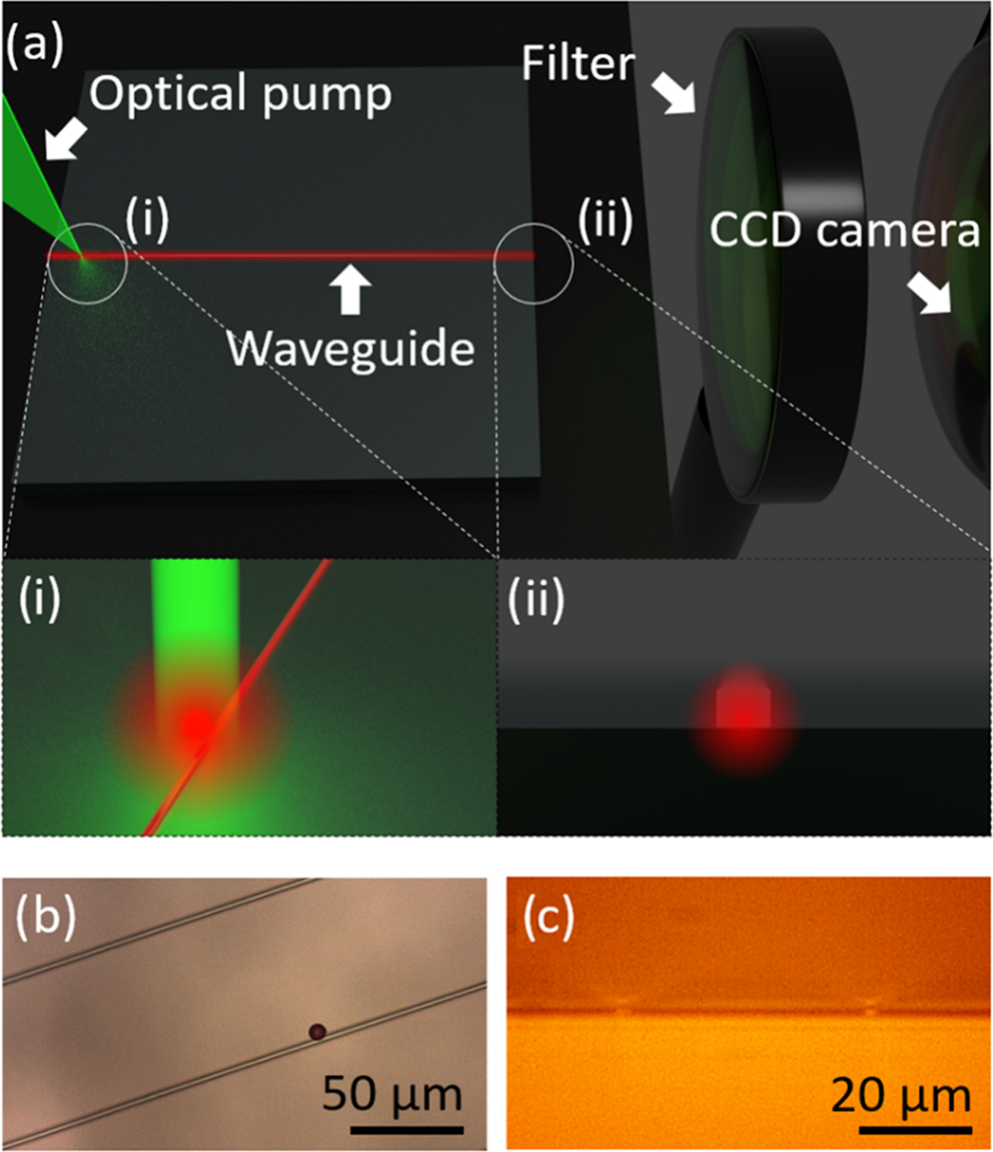
Illustration of the SP-waveguide coupling setup (a), where the sample is simultaneously aligned with the laser pump (a-i) and the CCD camera (a-ii). The SP (diameter ≈7.7 ± 0.5 μm) is being pumped on one edge of the waveguide, and the other edge the facet is being monitored by the CCD camera, which is preceded by a long pass filter (550 nm) to cut out any scattered light from the pump (λ_pump_ = 532 nm; see the Optical Characterization Section). The acquired microscope (b) and CCD camera (c) views correspond to the illustrations (a-(i, ii)), respectively. The spectrometer and image readings from the CCD camera were acquired simultaneously and compared to verify which modes were coupled to the waveguide. The full optical setup can be seen in Figure S7.

The process was first demonstrated by printing 15 SPs with average radii of 2.8 ± 1.7 μm from glass onto PDMS into a pattern following the shape of the University of Strathclyde logo (size distribution of SPs in Figure S2). Figure 4g,h displays the micrograph of the printed SPs under bright field and dark field with ultraviolet (UV) flooding conditions, respectively. All SPs are seen to luminesce after printing. This same process was then tested as a way to couple SPs to waveguides without affecting their capability as lasers. A rendered schematic (Figure 5a) summarizes the proof of concept experiment for the integration of an SP with a waveguide, where an SP is placed in contact with a waveguide, on its side, to enable evanescent field coupling between the two structures.

An SP (7.7 ± 0.5 μm in diameter) was transferred onto the silica surface and placed near one of the two facets of the waveguide with its surface in contact with the waveguide,^38^ as shown on the rendered inset (i) of Figure 5a. The stamp was then gently translated sideways and moved upward to release the SP. The light emitted by the SP and coupled in the waveguide was measured from the output facet at the other edge of the chip (rendered inset ii, Figure 5a), approximately 8 mm away. A second charged-coupled device (CCD) camera with a long pass filter (cutoff wavelength of 550 nm) was used to image the waveguide output facet through an objective lens. Figure 5b,c shows setup images of the SP under optical pumping and the end facet of the waveguide, respectively. The microscopic image in Figure 5b and the CCD camera view in Figure 5c are analogous to the rendered insets (i) and (ii). The full setup can be seen in Figure S7.

In dark-room conditions, the SP was excited at different pump energies (spot size of 4.85 × 10^–7^ cm^2^) and a micrograph of the waveguide output facet was acquired by the CCD camera for each of these energies, while the emission spectrum of the SP was recorded *via* the μPL setup simultaneously.

The signal-to-noise ratio (eq 6) is calculated from the pixel intensity of the images acquired by the CCD camera with the laser off (noise) and laser on (signal) within the region of interest, *i.e.*, the end facet of the waveguide (Figure S8)
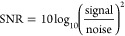
6

**Figure 6:**
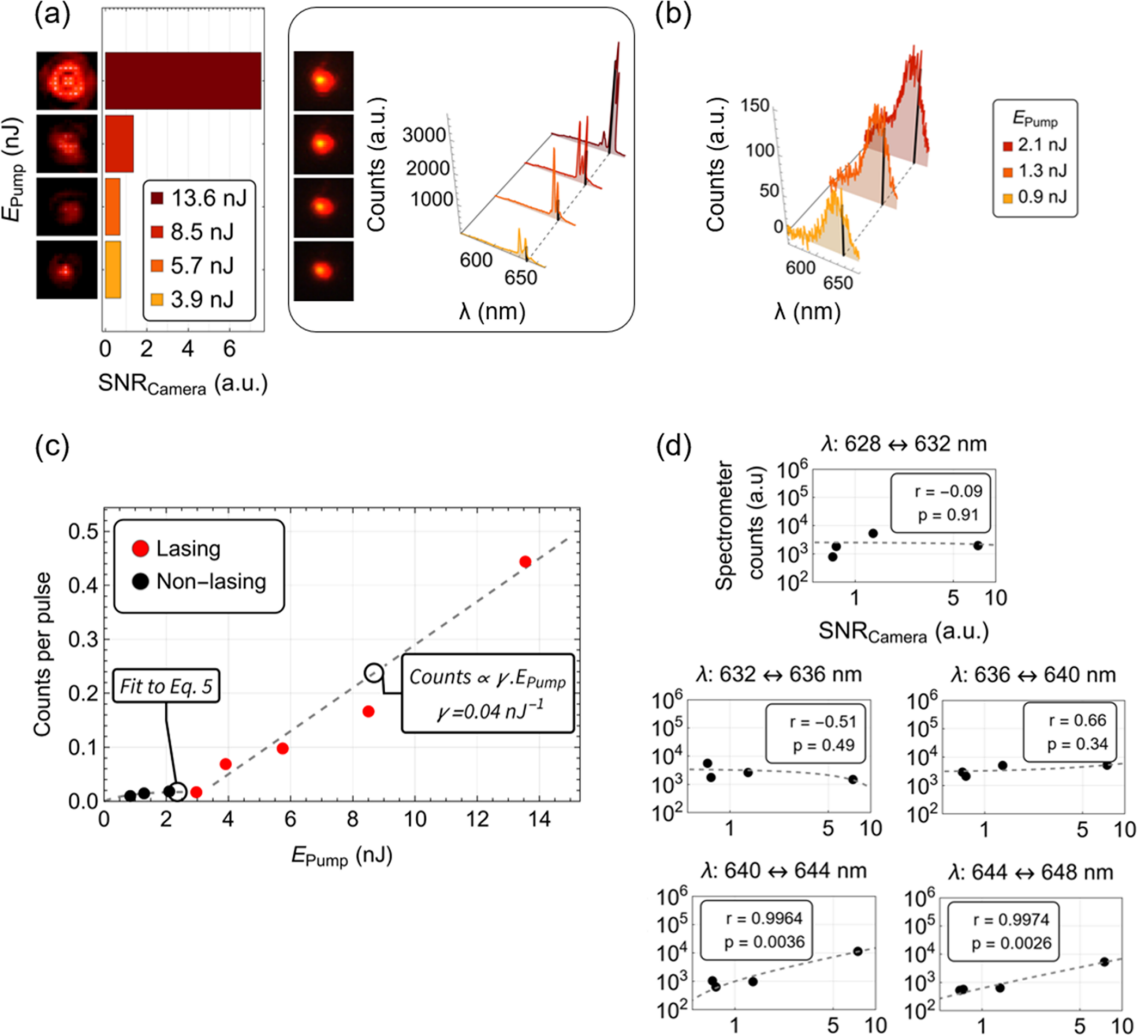
Readings of the CCD camera, with the enhanced facet pictures and corresponding data, depicted alongside the pictures of the transfer-printed SP (7.7 ± 0.5 μm in diameter) and readings on the spectrometer (a). These measurements were done under four different excitation intensities above the laser threshold. Spectrometer readings below a threshold and under three different excitation intensities are also shown in panel (b). The dashed line seen in spectra (a, b) tracks one of the modes of the SP at approximately 640 nm. The data acquired at that wavelength was used to plot the emission intensity versus pump energy (c), and the laser threshold of the transfer-printed SP was found to be at approximately 3 nJ. The spectral range where the lasing peaks were located in the SP (*i.e.*, from 628 to 648 nm) was divided into 5 equal parts of Δλ = 4 nm each. A Pearson correlation test between the counts registered on the CCD camera and the counts registered on the spectrometer was then performed on each of those 5 parts, using the data of the 4 different optical pump energies (d). The two test parameters, *r* and *p*, correspond to the Pearson correlation coefficient and *p*-value, respectively. SPs were optically pumped at λ_pump_ = 532 nm (see the Optical Characterization Section). The full optical setup can be seen in Figure S7.

The end facet data collected by the CCD camera and the corresponding spectral data acquired by the spectrometer can be seen in Figure 6a. Figure 6b complements the measurements of Figure 6a below the laser threshold. The laser transfer function based on the data of the spectra below and above the laser threshold can be seen in Figure 6c. From Figure 6c, the laser threshold occurs at approximately 3 nJ. This corresponds to an average number of excitons of approximately ⟨*N*⟩ = 1.7 (Figure S9), which is close to the values estimated prior to the transfer printing process (Figure 3b). The consistency between the average number of excitons before and after the transfer printing process is a strong indicator that this method can be reliably used to transfer SPs between substrates. The collected spectra were split in intervals of 4 nm over the range where modes oscillate (628–648 nm) to facilitate the comparison between the intensity at each interval and the signal-to-noise ratio (SNR). This comparison is complemented by a Pearson correlation test to study the relationship between these two types of measurement (Figure 6d). The test is assessed based on two test parameters, the Pearson correlation coefficient (*r*), and the *p*-value (*p*). The Pearson correlation coefficient measures the linear correlation between the two sets of data (−1 ≤ *r* ≤ 1), and the *p*-value gives the probability of obtaining test results that are at least as extreme as the result actually observed, under the assumption that the null hypothesis is correct (0 ≤ *p* ≤ 1). If the null hypothesis of this test is established as the linear independence between the readings on the CCD camera and a given spectral range on the spectrometer, the results show that the null hypothesis is not rejected at the ≤5% level between the 628 and 640 nm (Figure 6d). However, for the longest wavelength modes (640–648 nm), the same null hypothesis is rejected at ≤5%. This indicates that the waveguide output at the end facet is strongly correlated (>95% confidence) to the longer-wavelength laser modes of the SP and therefore indicates that these long-wavelength modes are preferentially coupled into the waveguide (Figure 6d). This behavior can be explained by the geometry of the system and the location of the different WGMs in the SP. The diameter of the SP (7.7 ± 0.5 μm) is bigger than the cross section of the waveguide (2 μm × 2 μm). Once the SP reaches the laser threshold, the first modes are likely confined to the equatorial region of the SP. However, higher pump energies enable higher azimuthal modes to oscillate, in this case with longer wavelengths, that are more easily coupled to the waveguide.

## Summary and Conclusions

A current barrier to the use of colloidal nanocrystal SP lasers in integrated optics is the lack of a controllable and scalable technique for the deterministic manipulation of such microlasers. In this work, microtransfer printing has been proposed and demonstrated as a solution to this problem. CdS_*x*_Se_1–*x*_/ZnS CQD SPs with diameters ranging from 4 to 20 μm and Q-factors of approximately 300 were self-assembled *via* an oil-in-water process. SPs with sizes below 10 μm in diameter achieved lasing on the μPL setup between 625 and 655 nm, with one or several angular modes oscillating depending on the pump level and the SP. Using the proposed model for the emission of SPs below the laser threshold, the average number of excitons ⟨*N*⟩ at the threshold was found to be ⟨*N*⟩ = 2.6 ± 0.8, a value that is consistent with the ⟨*N*⟩ = 2.5 simulated for SPs of CQDs.^13^ This model could help in the assessment of enhancements of SPs by studying the evolution of its parameters. The microtransfer printing process demonstrated the pickup and drop off of individual SPs onto different substrates without affecting their laser capability. As a proof of concept, an SP laser was integrated and side-coupled to a polymer waveguide. The laser threshold fluence of the transfer-printed SP (≈6.2 mJ.cm^–2^) is within the range of laser threshold fluences of SPs studied in Figure 3 (2.7–19.1 mJ.cm^–2^). The fitted parameters give an estimated average number of excitons for the transfer-printed SP of ⟨*N*⟩ ≈ 1.7, which is also close to the values of SPs studied in Figure 3, with ⟨*N*⟩ = 2.6 ± 0.8. Under optical pumping, specific laser modes of the SP were successfully coupled and detected at the end of the waveguide facet. This makes the transfer printing method a strong contender for future integrated photonic applications of SPs and paves the way to more complex designs.

## Materials and Methods

### Chemicals

Cadmium selenide sulfide (core), zinc sulfide (shell), and trilite fluorescent CQDs, with alkyl ligands, were ordered from Cytodiagnostics (Cytodiagnostics, Canada). Chloroform (anhydrous, 99.5%) and poly(vinyl alcohol) (average *M*_w_ 85,000–124,000, 87–89% hydrolyzed) were ordered from Merck and used as received. Water was purified with a Milli-Q water purification system.

### Synthesis and Characterization of the SPs

The self-assembly of SPs followed an oil-in-water emulsion prepared at room temperature. Two immiscible solutions were prepared, one with the CQDs dissolved in chloroform at a concentration of approximately 250 mg/mL and another with PVA dissolved in Milli-Q water at a mass ratio of 1.25%. The emulsion was prepared by vortexing 115 μL of the CQD solution with 450 μL of the water solution for 10 min and stirring the mixture for approximately 2 h at 750 rpm. Once the stirring was completed, self-assembled SPs were diluted in water at a volume ratio of 1:50 and vortexed again to remove traces of PVA on their surfaces. Samples in this work were prepared by drop-casting 10 μL of cleaned SPs onto a glass substrate. Their size distribution and polydispersity can be seen in Figure S2.

### Microfabrication and Characterization of Waveguides

Waveguides were fabricated by laser lithography. A 2 μm layer of SU-8 2 (MicroChem), with a viscosity of 45 cSt, was spin-coated (30 s at 2000 rpm) onto a pretreated glass substrate. The pretreatment of the glass included a 10 min ultrasonic bath in acetone and a 10 min ultrasonic bath in isopropanol before it was rinsed in water and dried. The SU-8-coated glass substrate was soft-baked 1 min at 65 °C and 3 min at 95 °C. The photolithography step to pattern the waveguides was done with a custom maskless laser lithography tool (λ = 370 nm). After photolithography, the sample was baked again for 1 min at 65 °C and 1 min at 95 °C. The postexposure baking was followed by the development of the SU-8 resist for 1 min, using a MicroChem’s SU-8 Developer. The waveguides were cleaved on both ends. These ended with 8.0 ± 0.5 mm in length and with a cross section of 2 μm × 2 μm, which makes them multimode at the wavelengths of interest (visible light spectrum) with an increased numerical aperture (NA) for input coupling and compatible with several optical interconnect techniques.^39^ The propagation losses were measured with a narrow linewidth tunable laser (λ = 1550 nm). The propagation loss of the waveguides was measured using the fast Fourier transform (FFT) method and estimated to be less than 3 dB·cm^–1^ for the fundamental mode.^40^

### Transfer Printing the SPs

A polymer μ-stamp of poly(dimethylsiloxane) (PDMS) was made from elastomer and curing agent at a 10:1 ratio (SYLGARD 184 Silicone Elastomer Kit) and cured at 60 °C. The stamp was then used in a modified dip-pen nanolithography system to pick up SPs (Figure 4). SPs were moved with submicron resolution from the substrate where they were initially drop-casted to the substrate with the waveguide.^41^ An auxiliary camera embedded in the system allows the user to control the transfer printing process.^42^

### Optical Characterization

The PL and absorbance spectra of CQDs can be found in Figure S1. The SPs were optically pumped with a 0.76 ns pulse width microchip pulsed laser (λ = 532 nm, MNG-03 × 10^–100^, Teem Photonics) at a repetition rate of 7.1 kHz and with a beam spot area of approximately 2.88 × 10^–7^ cm^2^ for the individual characterization and 4.85 × 10^–7^ cm^2^ for the coupling. The beam was attenuated with a variable wheel attenuator and focused on the sample with an objective lens (4×, NA0.13, Nikon). A spectrometer (AvaSpec-2048–4-DT, Avantes) with a 0.7 nm spectral resolution between 220 and 1100 nm was used to acquire the spectrum data. A power meter was used to calibrate the output energy as a function of the attenuator filter before each experiment. More details on the setup can be seen in Figure S3.

### SP-Waveguide Coupling

To visualize if light was being coupled on waveguides, an extra camera (DCC1645C, Thorlabs) was installed on the μPL setup for this experiment (Figure S7). Measurements were taken in the dark. A long pass filter (FEL0550, Thorlabs) was attached to the camera to cut off stray light from the pump. SPs were coupled and pumped on one end of the waveguide and the camera was focused on the facet of the other end to image the light coupled to it. Images were acquired and processed at different pump fluences.

### SEM Characterization

SPs were also characterized by an FEI Quanta 250FEG scanning electron microscope (SEM). A custom-built CL setup collects light perpendicular to the beam excitation through a reflecting objective. The spectrum is measured using a 0.125 m spectrometer containing a 50 μm slit and a 600 lines/mm grating, paired with a cooled back-illuminated electron multiplying charge-coupled device.^43^ Elemental analysis was performed on SPs through energy-dispersive X-ray spectroscopy (EDS) to map and visualize the elements present.

## Supporting Information Available

Quantum dots (1); size distribution of supraparticles (2); design of the microphotoluminescence setup for characterization of supraparticles (3); electric field in a microsphere and whispering gallery modes of supraparticles (4); characterization of supraparticles (5); design of the microphotoluminescence setup for characterization of coupled devices (6); signal-to-noise processing (7); and characterization of the transfer-printed supraparticle (PDF)

## Abbreviations

NC: nanocrystal
SP: supraparticle
WGM: whispering gallery mode
QD: quantum dot
CQD: colloidal quantum dot
*Q*-factor: quality factor
CQW: colloidal quantum well
PVA: poly(vinyl alcohol)
PL: photoluminescence
SEM: scanning electron microscope
CL: cathodoluminescence
FSR: free spectral range
PDMS: poly(dimethylsiloxane)
CCD: charged-coupled device
UV: ultraviolet
SNR: signal-to-noise ratio
NA: numerical aperture
