# Supplementary material for: Waveguide-Integrated Colloidal Nanocrystal Supraparticle Lasers
Source: ACS Appl Opt Mater. 2023 Nov 15;1(11):1836–46. doi: 10.1021/acsaom.3c00312 (PMC10683367; doi:10.1021/acsaom.3c00312)
Supplement: Supplementary file 1 — ot3c00312_si_001.pdf [file ot3c00312_si_001.pdf]

Supporting Information

Waveguide-Integrated Colloidal Nanocrystal Supraparticle Lasers

Pedro Urbano Alves, Benoit J. E. Guilhabert, John R. McPhillimy, Dimitars Jevtics, Michael J. Strain, Matěj Hejda, Douglas Cameron, Paul R. Edwards, Robert W. Martin, Martin D. Dawson, Nicolas Laurand.*

Institute of Photonics, Department of Physics, SUPA, University of Strathclyde, Technology and Innovation Centre, 99 George Street, Glasgow, G1 1RD, UK

Department of Physics, SUPA, University of Strathclyde, John Anderson Building, 107 Rottenrow, Glasgow, G4 0NG, UK

*email address: pedro.alves@strath.ac.uk

Quantum Dots. Supraparticles (SPs) synthesized in this work used Trilite™ fluorescent QDs (Cytodiagnostics, Canada), with Alkyl ligands.

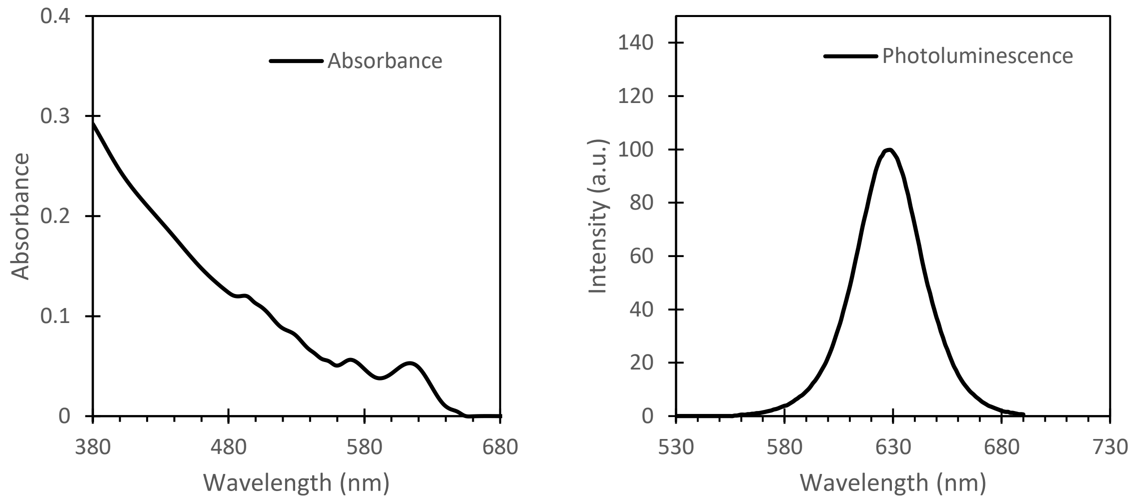

Figure S1. Absorbance (left) and photoluminescence (right) spectrums of the used commercial QDs. The photoluminescence was measured using a blue LED as the excitation source ($\lambda_{\mu LED}$ = 440 nm). The emission peak of the CQDs was measured at 628.1 nm. FWHM was approximately 38 nm.

These QDs have a CdS_xSe_{1-x} core and a ZnS shell. Additional data from the supplier include: the average diameter of nanocrystals, which ranges between 5.5 and 6.5 nm; absorption spectrum and emission spectrum with a peak of 630 ± 5 nm and a full width at half maximum (FWHM) of

30-40 nm. Figure S1 shows the absorbance and photoluminescence spectrums of these nanocrystals.

Size distribution of supraparticles. To characterize the size of SPs, after the self-assembly process, a sample solution of synthesized SPs was drop cast on glass and observed under the microscope. Figure S2 shows the scaled picture of SPs (left) and the histogram of their measured radius (right). The radius was measured automatically using a custom-made algorithm on Wolfram Mathematica software. The algorithm follows steps similar to others already reported, [1] combined with image processing functions from the software. A sample of SPs measured this way (N = 221) had an average radius of 2.8 µm ± 1.7 µm and a polydispersity of approximately 60%. The polydispersity (PD) is given by $PD = \frac{r_\sigma}{r_\mu}.100\ \%$, where r_μ is the average radius of SPs and r_σ is their standard deviation.

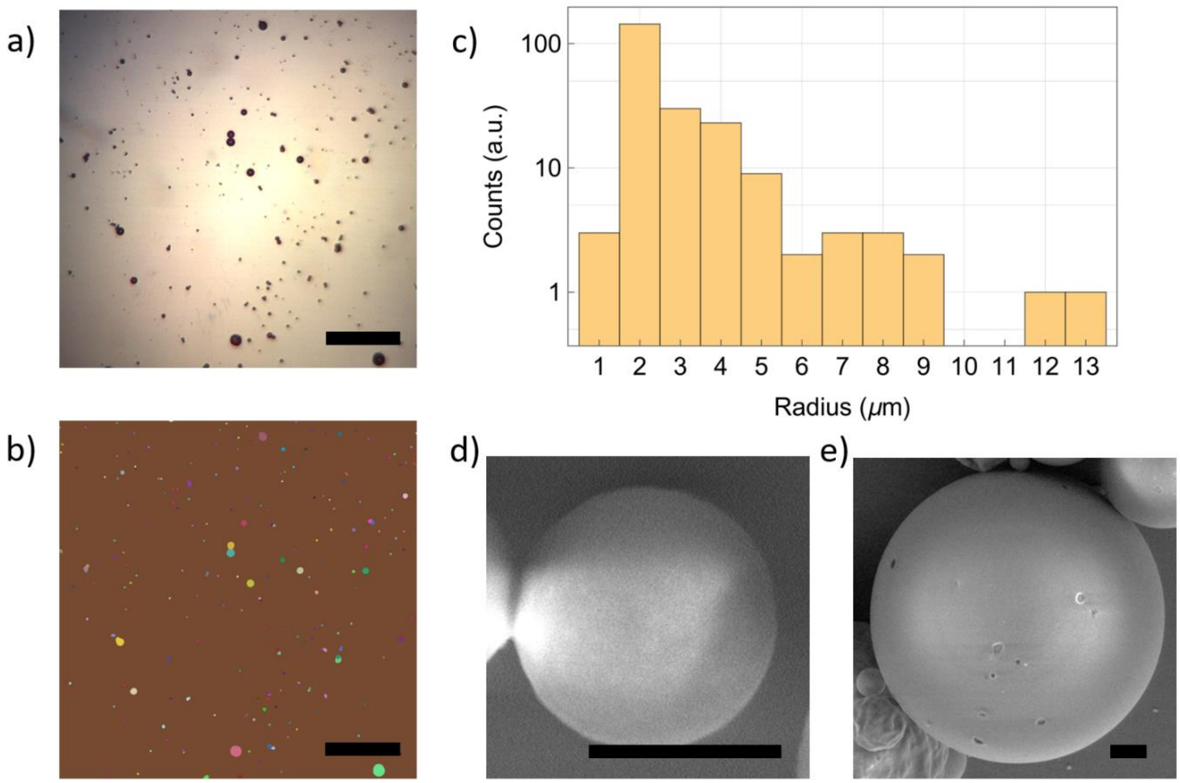

Figure S2. Estimation of the size distribution of SPs from a microscope image (a). The Otsu's method was used in combination with the "watershed components", "select components" and "component measurements" functions in Wolfram Mathematica to separate foreground and background and automatically identify, count and measure the SPs (b). The scale bars in (a) and (b) correspond to 180 µm. The distribution of SPs according to their radius is shown in the histogram (c). The average radius size of SPs in this sample was 2.8 µm ± 1.7 µm (sample size:

221 SPs). Examples of small and large SPs seen on SEM are illustrated in (d) and (e), respectively. The scale bars of the SEM images (d) and (e) correspond to 3 μm.

Design of the μ-Photoluminescence setup for characterization of supraparticles. The μ-Photoluminescence (μ-PL) design used to characterize SPs is represented in Figure S3.

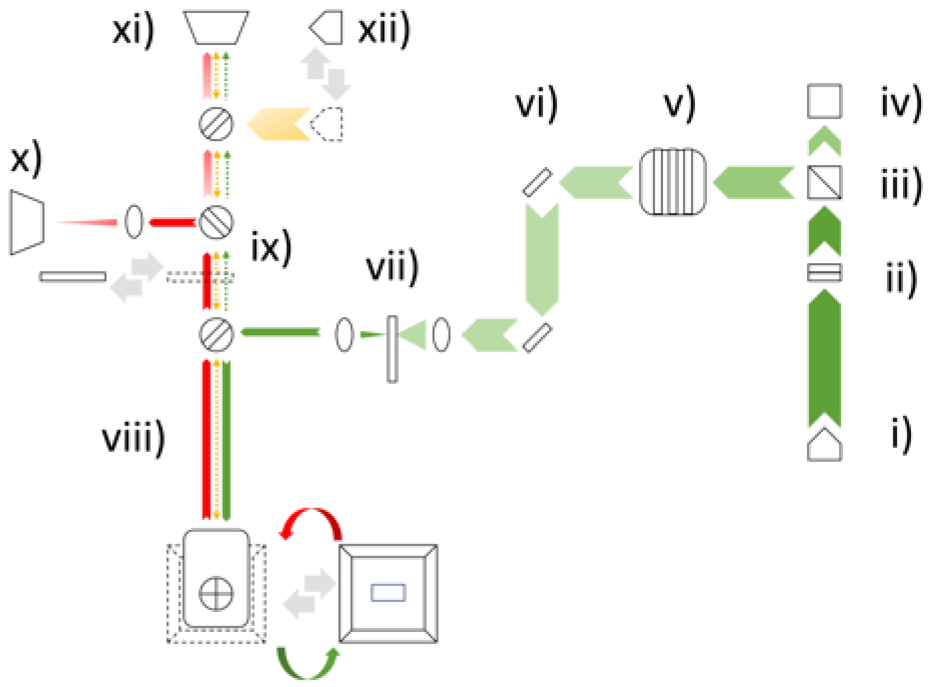

Figure S3. Schematic of the μ-Photoluminescence setup: i) Pump source (532 nm laser); ii) Waveplate; iii) Polarised beam splitter; iv) Beam dump; v) set of neutral density filters; vi)

mirrors; vii) beam expander with attenuator wheel incorporated; viii) setup with a mounted objective lens (4×/0.13) and a xyz stage where the sample is placed; ix) set of 3 beam splitters and a long pass filter (550 nm); x) Spectrometer fiber-coupled to the setup; xi) CCD camera; xii) lamp. Green arrows represent the path of the laser from the pump, red arrows represent the path of the laser from the SP and yellow arrows represent the light path of the lamp.

This setup is based on a previous design [2] and operates with a 0.76 ns pulse width microchip pulsed laser (λ = 532 nm) at a repetition rate of 7.1 kHz and with a beam spot area of approximately 2.88×10^{-7} cm^2. The energy of this laser is attenuated by a waveplate ($\lambda/2$), polarizer and neutral density filter. This setup uses a custom-made beam expander with two plano-convergent lenses (focal lengths f1 = 12.5 cm and f2 = 2.5 cm). An attenuator wheel is incorporated within the beam expander to allow fine control of the pump intensity during experiments. A set of beam splitters is used to guide light into an objective lens (4× magnification; numerical aperture, NA = 0.13), in order to focus the pump light onto the sample. At focus, the beam spot size had a diameter of 6 µm. Samples are placed on a xyz stage that gives full control on their positioning. The light emitted by

the sample is collected by the same objective and sent via a set of beam splitters onto the fiber coupled spectrometer (AvaSpec-2048-4-DT with 0.7 nm of spectral resolution between 220-1100 nm using an optical fiber with a core of 50 μm in diameter, Avantes) and CCD camera (DCC1645C, Thorlabs). A long pass filter (FEL0550, Thorlabs) is placed between the first two beam splitters when needed (either during the acquisition of measurements or camera capture). A lamp, also coupled to the set of beam splitters, can be switched on to help visualizing features on camera.

Electric field in a microsphere and whispering gallery modes of supraparticles. The calculations of the Transverse Electric (TE) and Transverse Magnetic (TM) electric modes for SPs in this work were based on the method written by Stéphane Balac and Patrice Feron (Stéphane Balac, Patrice Feron. "Whispering gallery modes volume computation in optical micro-spheres". [Research Report] FOTON, UMR CNRS 6082. 2014. Hal-01279396v2), and Wolfram Mathematica™ was used to calculate and plot the WGMs.

The modal equation for TE electric field modes in a microsphere obtained by the Maxwell equations is given by:

$$\underline{\boldsymbol{E}}(r,\theta,\varphi) = A_{i/o}^{TE} \frac{r_l(kr)}{kr} \boldsymbol{X}_{lm}(\theta,\varphi) \qquad \text{(Eq. S1)}$$

Where $A_{i/o}^{TE}$ is a term whose value depends on the domain (index i when inside the SP and index o when outside the SP):

$$\begin{cases} A_i^{TE} = 1 \\ A_o^{TE} = -A_i^{TE} \frac{k_0}{k_0 N} \frac{\psi(l,k_0 NR)}{\zeta(l,k_0 R)} \end{cases} \qquad \text{(Eq. S2)}$$

With ψ being related to the Ricatti-Bessel function of first kind (uses "$SphericalBesselJ$[ ]" in Wolfram Mathematica™):

$$\psi(l,z) = z.SphericalBesselJ[l,z] \qquad \text{(Eq. S3)}$$

And ζ to the Ricatti-Bessel function of third kind:

$$\zeta(l,z) = \psi(l,z) + i\chi(l,z) \qquad \text{(Eq. S4)}$$

Where χ is associated to Ricatti-Bessel function of second kind (uses "$SphericalBesselY$[ ]" in Wolfram Mathematica™):

$$\chi(l,z) = -z.SphericalBesselY[l,z] \quad \text{(Eq. S5)}$$

The wavenumber inside the SP, k, depends on the refractive index of the SP, N, and on the wavenumber in vacuum, k_0:

$$k = k_0 N; k_0 = \frac{2\pi}{\lambda_0} \quad \text{(Eq. S6)}$$

The radial term r_l also depends on the domain:

$$r_l(kr) = \begin{cases} \psi(l, k_0 N r), 0 \leq r \leq R \\ \zeta(l, k_0 r), r > R \end{cases} \quad \text{(Eq. S7)}$$

Where R is the radius of the microsphere.

$X_{lm}(\theta,\varphi)$ corresponds to one of the three vector spherical harmonics ($X_{lm}(\theta,\varphi)$, $Y_l(\theta,\varphi)$, $Z_{lm}(\theta,\varphi)$) and is given by the wedge product between the gradient of the scalar spherical harmonics and the vector $\boldsymbol{r} = \{r, 0,0\}$:

$$\boldsymbol{X}_{lm}(\theta,\varphi) = \nabla Y_l^m \wedge \boldsymbol{r} \quad \text{(Eq. S8)}$$

The scalar spherical harmonics is given by a Wolfram Mathematica™ function:

$$Y_l^m = SphericalHarmonicY[l,m,\theta,\varphi] \quad \text{(Eq. S9)}$$

And the gradient ∇Y_l^m can be calculated by Wolfram Mathematica™ using the "$Grad[\,]$" function:

$$\nabla Y_l^m = Grad[SphericalHarmonicY[l,m,\theta,\varphi],\{r,\theta,\varphi\},"Spherical"] \qquad \text{(Eq. S10)}$$

Likewise, the modal equation for TM electric modes is given by:

$$\underline{\boldsymbol{E}}(R,\theta,\varphi) = A_{i/o}^{TM}\left(l(l+1)\frac{r_l(kR)}{k^2R^2}\boldsymbol{Z}_{lm}(\theta,\varphi) + \frac{r_l{'}(kR)}{kR}\boldsymbol{Y}_{lm}(\theta,\varphi)\right) \qquad \text{(Eq. S11)}$$

With:

$$\begin{cases} A_i^{TM} = 1 \\ A_o^{TM} = -A_i^{TM}\frac{\psi(l,k_0NR)}{\zeta(l,k_0R)} \end{cases} \qquad \text{(Eq. S12)}$$

$$r_l(kr) = \begin{cases} \psi(l,k_0Nr)\,, 0 \le r \le R \\ \zeta(l,k_0r)\,, r > R \end{cases} \qquad \text{(Eq. S13)}$$

$$r'_l(z) = \begin{cases} \frac{d\psi(l,z)}{dz}\,, 0 \le r \le R \\ \frac{d\zeta(l,z)}{dz}\,, r > R \end{cases} \qquad \text{(Eq. S14)}$$

The other two vector spherical harmonics, $\boldsymbol{Y}_{lm}(\theta,\varphi)$ and $\boldsymbol{Z}_{lm}(\theta,\varphi)$, are given by:

$$\boldsymbol{Y}_{lm}(\theta,\varphi) = r\nabla Y_l^m \qquad \text{(Eq. S15)}$$

$$\boldsymbol{Z}_{lm}(\theta,\varphi) = Y_l^m \boldsymbol{e_r} = Y_l^m.\{1,0,0\} \qquad \text{(Eq. S16)}$$

The expected WGMs for a SP with 9.8 ± 0.5 μm in diameter were calculated numerically and compared to the WGM pattern observed under the microscope (Figure S4). The WGMs are characterized by three parameters referred to as n, l and m [24], which correspond, respectively, to the number of maxima of the radial, angular and azimuthal field distribution. For this numerical experiment, the sphere was of the same diameter as the SP observed under the microscope and had a refractive index of N = 1.7. [3] First, the modal equations for the electric field (transverse electric – TE, and transverse magnetic – TM) [4] were solved for a wavelength close to the observed lasing peaks (λ = 636 nm) in order to determine l for which the resonances occur (Figure S4a for TE modes and Figure S4b for TM modes).

Once the values of l are known, the exact resonance wavelengths located within the laser emission range observed in the PL spectrum of the SP can then be found using the modal equations, this time using the l values determined above and finding the wavelengths for which the modal equations are equal to zero (Figure S4b for TE modes and Figure S4d for TM modes). Table S1

shows the resonance wavelengths found for this numerical experiment in the region of interest, i.e. the wavelength range for the lasing peaks of Figure S4i.

Table S1. Radial index, angular field distribution and resonant wavelengths solved in the region of interest (wavelength close to the observed lasing peaks, *i.e.* λ = 636 nm) and for a SP with a diameter 9.8 µm and refractive index of N = 1.7.

	TE		TM	
n	l	λ (nm)	l	λ (nm)
1	75	636	74	635
2	69	636	68	638
3	64	638	63	640
4	60	635	59	638

5	56	637	56	632
6	53	632	52	637
7	49	637	49	635

The spacing between several resonant wavelengths falls below the resolution of the spectrometer (Methods – Optical Characterization), and therefore it is difficult to identify the exact TE and TM modes. Nevertheless, the range of resonant frequencies found in this numerical experiment (mainly between 635 and 640 nm) match those observed experimentally (Fig. 2i). Small deviations between the numerical and laboratorial measurements can be assigned to the not perfectly spherical shape of the SPs and small fluctuations in the refractive index.

Once the parameters above are found, the real component of the electric field normalized for TE and TM modes can be evaluated and plotted (Equations. S1 to S16).

Figures S4e – h illustrate such intensity maps in the xy and xz plane for a TE (n = 1, l = 75, λ = 636 nm, m = 20; Figure S2e and S2g) and TM mode (n = 1, l = 74, λ = 635 nm, m = 20;

Figure S2f and S2h). These WGMs were solved numerically and correspond to travelling mode solutions. Therefore, they cannot be directly compared with the imaged vertical scatter from the steady-state image (Figure S4j). The latter, since it is captured in steady state on the camera is not a travelling wave – where wavefronts would propagate around the sphere – but is rather a standing wave, likely formed by degenerate counter-propagating modes.

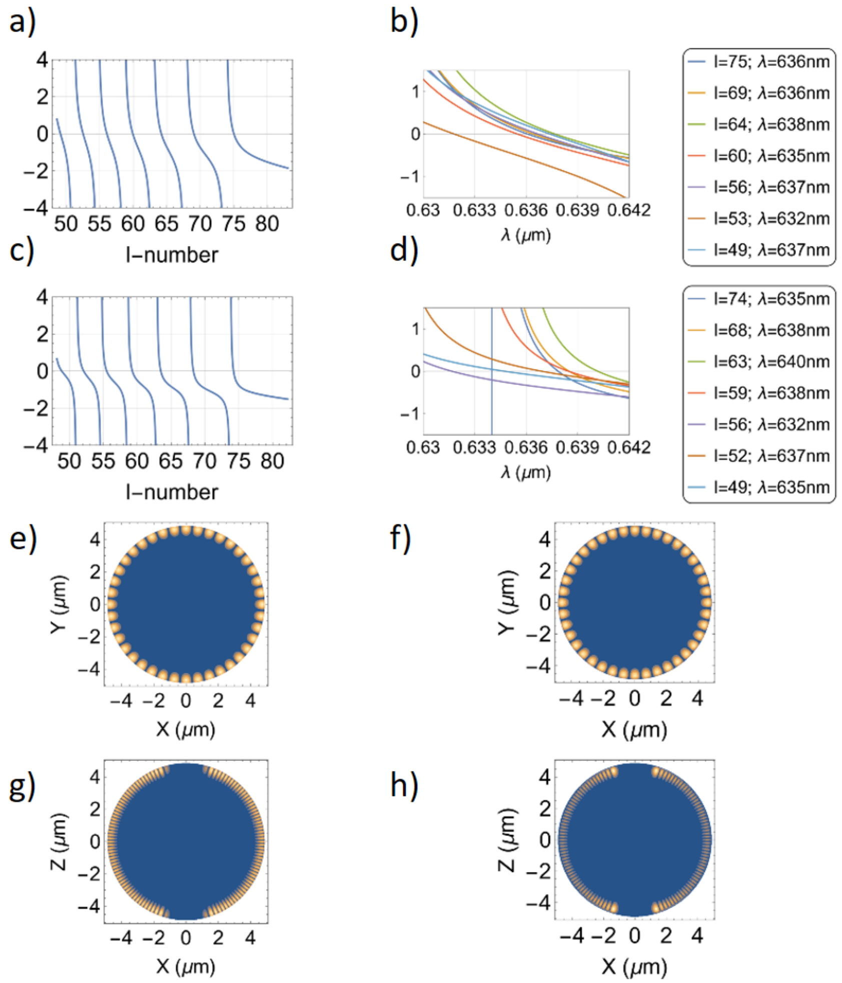

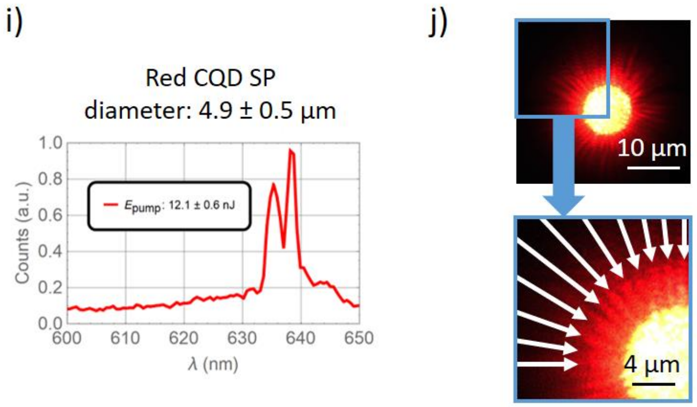

Figure S4. Study on the modes of a SP. The mode number l and resonant frequencies were calculated by solving the modal equations [4] for TE (a; b) and TM fields (c; d). The numerical experiment considers a SP with the same diameter as the one measured experimentally (9.8 µm) and a refractive index of 1.7. [3] These results were checked for consistency with the lasing peaks seen in the spectrum measured experimentally with the optical pump at 12.1 nJ (i). Modal intensity plotted in the xy-plane and xz-plane for both TE (e and g: n = 1, l = 75, λ= 636 nm, m = 20) and TM (f and h: n = 1, l = 74, λ = 635 nm, m = 20) had the spatial periodicity of their maxima compared to those seen experimentally (j). The SPs in the micrographs were optically pumped at λ_{pump} = 532 nm. The full optical setup can be seen in Figure S3.

It could in fact be a complex superposition of multiple counterpropagating mode orders and therefore not directly equatable with the calculated WGM solutions. For higher radial field distribution numbers ($n > 1$) evidenced in Table S1 and by the two broad lasing peaks in Figure S4i, which match the resonant wavelengths calculated in Figures S4b and S4d (for TE and TM modes, respectively), the mode volumes expand towards the center of the SP regardless of the high absorption coefficient found in CQDs [3]. The exact number of lasing modes and their quantum numbers are dependent on the pump energy, the pump spot alignment with the SP, and the size of the latter.

Characterization of SPs. Table S2 shows a compilation of the 5 SPs from Figure S5 that showed lasing, together with their PL spectrum at different energies (below and above lasing threshold), estimated threshold energies, radius and average number of excitons in the SP.

Table S2. List of the SPs that went above the lasing threshold for the range of energies studied in this work, together with their PL spectra, laser transfer functions (detected counts per pulse at the

following resonant wavelengths: 633 nm, 634 nm, 644 nm, 634 nm, 640 nm. These resonant wavelengths are for SP nr 1, 3, 4, 5 and 15, respectively) and main parameters (*i.e.* size and approximate ⟨N⟩ at laser threshold). SPs were optically pumped at λ_{pump} = 532 nm. The full optical setup can be seen in Figure S3.

<table>
<tr><th>SP (nr)
&
Radius
(μm)</th><th>PL</th><th>Laser transfer function</th><th>E_{pump} at
threshold
(nJ) &⟨N⟩ at
threshold</th></tr>
<tr><td>1;
3.0 ± 0.5</td><td>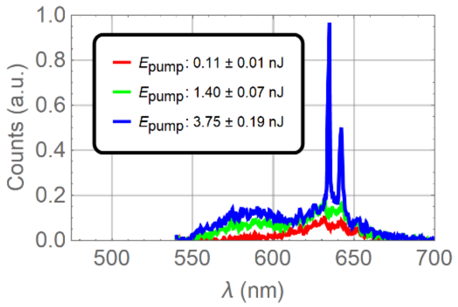
</td><td>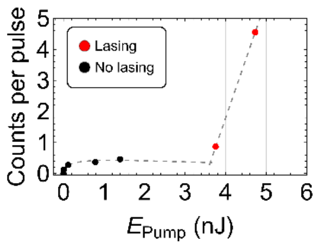
</td><td>2.6 ± 1.1;
1.3</td></tr>
<tr><td>3;
2.0 ± 0.5</td><td>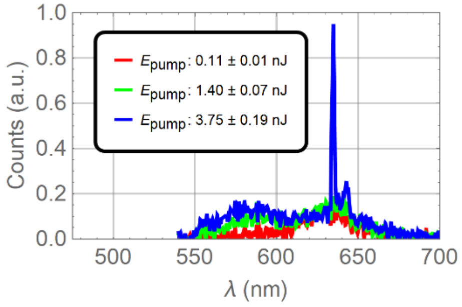
</td><td>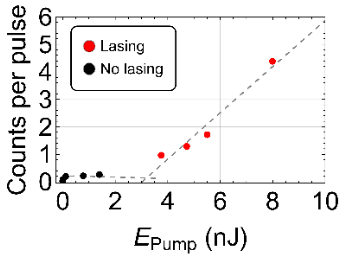
</td><td>2.6 ± 1.1;
2.8</td></tr>
</table>

4; 2.4 ± 0.5

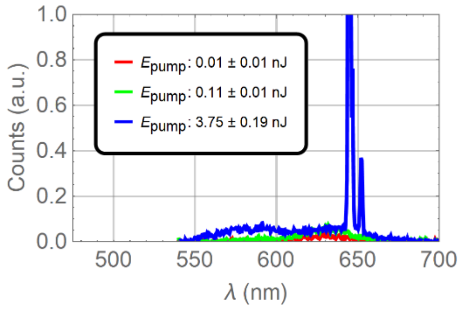

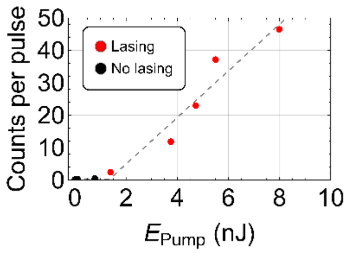

1.1 ± 0.3; 2.6

5; 3.2 ± 0.5

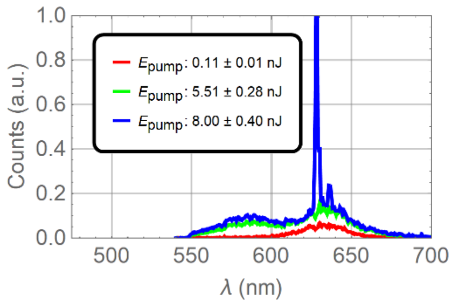

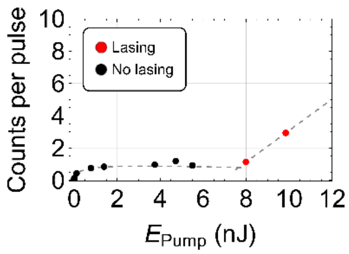

6.8 ± 1.2; 1.6

15; 4.9 ± 0.5

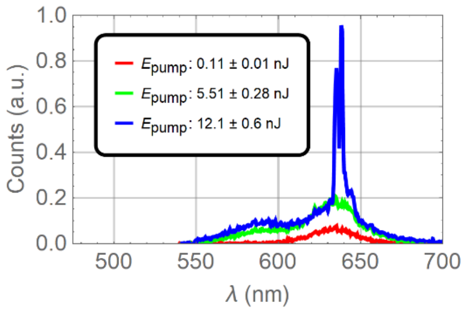

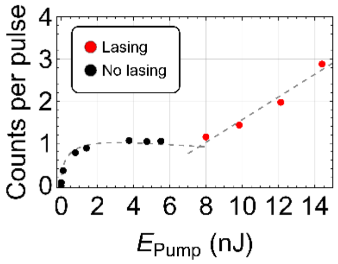

6.8 ± 1.2; 2.4

The fits of the sixteen SPs randomly picked can be seen in Figure S5, together with their sizes and inset fit results. The energy readings of the beam were spread over a gaussian function and integrated over the cross section of each SP to estimate the incident energies in Figure S5.

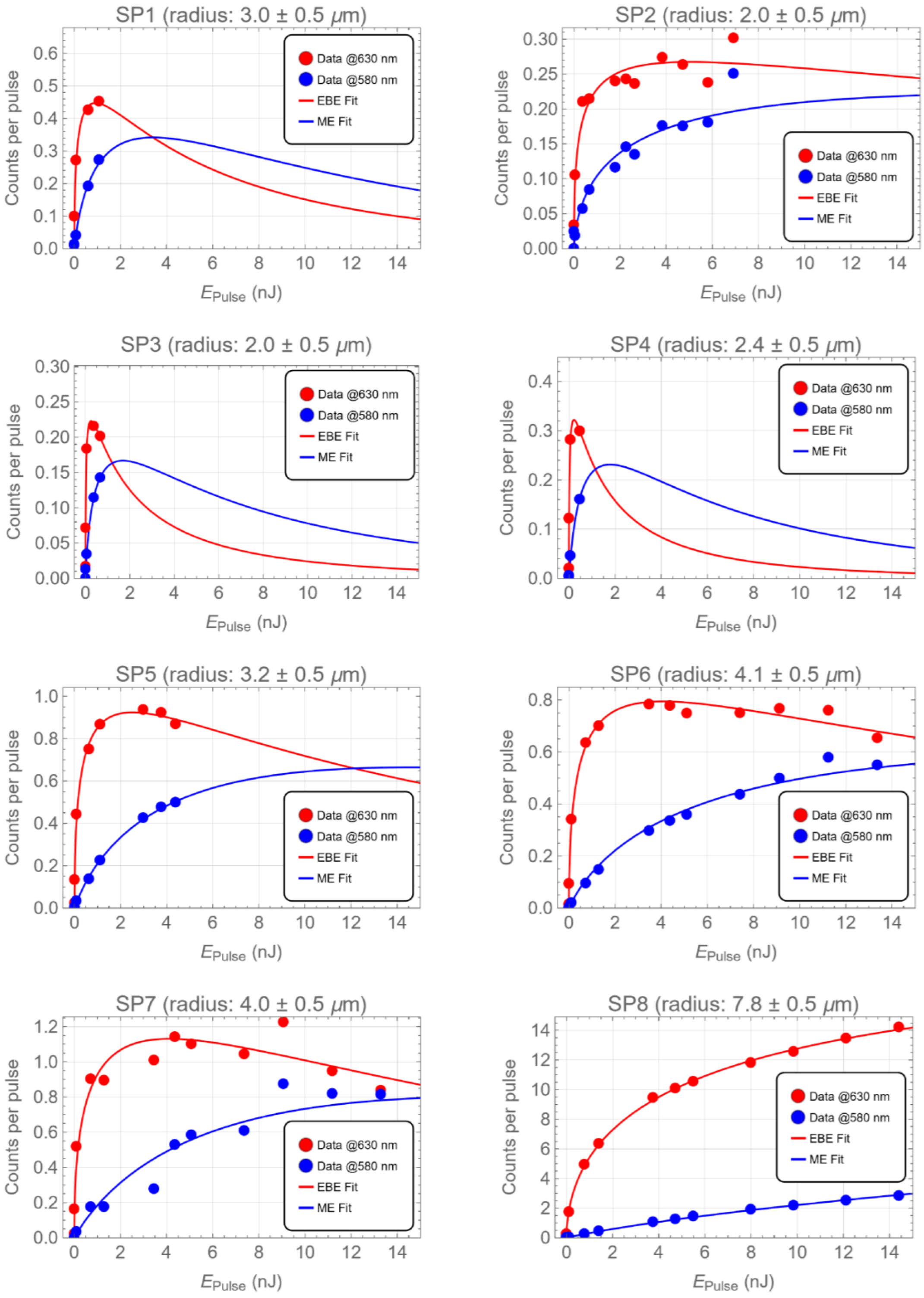
SP1 (radius: 3.0 ± 0.5 μm)
SP2 (radius: 2.0 ± 0.5 μm)
SP3 (radius: 2.0 ± 0.5 μm)
SP4 (radius: 2.4 ± 0.5 μm)
SP5 (radius: 3.2 ± 0.5 μm)
SP6 (radius: 4.1 ± 0.5 μm)
SP7 (radius: 4.0 ± 0.5 μm)
SP8 (radius: 7.8 ± 0.5 μm)
Data @630 nm
Data @580 nm
EBE Fit
ME Fit
Counts per pulse
E_{Pulse} (nJ)

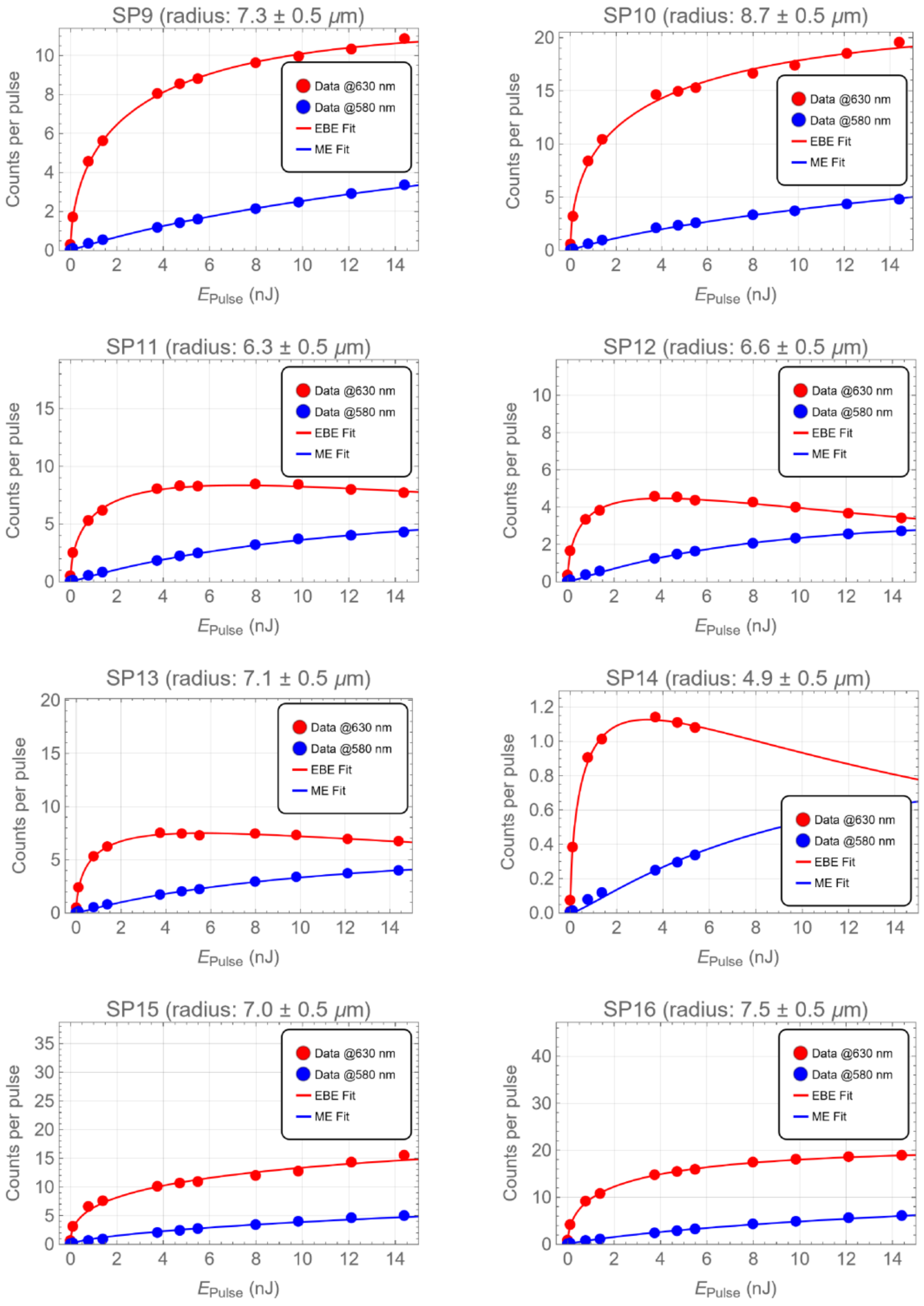
SP9 (radius: 7.3 ± 0.5 μm)
SP10 (radius: 8.7 ± 0.5 μm)
SP11 (radius: 6.3 ± 0.5 μm)
SP12 (radius: 6.6 ± 0.5 μm)
SP13 (radius: 7.1 ± 0.5 μm)
SP14 (radius: 4.9 ± 0.5 μm)
SP15 (radius: 7.0 ± 0.5 μm)
SP16 (radius: 7.5 ± 0.5 μm)
Counts per pulse
E_{Pulse} (nJ)
Data @630 nm
Data @580 nm
EBE Fit
ME Fit

Figure S5. Fit of Equation 5 to the exciton/biexciton peaks ($\bar{k}_1$) and multiexciton peaks ($\bar{k}_2$) for SPs of different sizes.

For simplification, Mie theory was neglected in this approach as the size of SPs are larger than the pump wavelength.

Table S3. Fitted parameters to the experimental data in Fig. S5 using Eq. 5.

SP nr	a	b	α	k_1 (nm)	k_2 (nm)
1	4.8±1.8 $\times 10^{-2}$	5.8±1.2	4.6±2.6 $\times 10^{-1}$	1.2±1.0	2.2±1.5
2	1.2±1.3 $\times 10^{-1}$	2.3±4.7	2.6±3.7 $\times 10^{-1}$	2.0±4.6	3.0±5.8
3	1.0±0.4 $\times 10^{-1}$	6.2±1.9	3.2±1.8 $\times 10^{-1}$	2.0±1.8	3.7±2.6
4	8.6±4.2 $\times 10^{-2}$	8.0±1.7	3.4±2.3 $\times 10^{-1}$	2.0±2.2	4.0±3.3
5	9.0±1.5 $\times 10^{-2}$	3.6±0.5	4.1±1.0 $\times 10^{-1}$	1.3±0.5	2.7±0.8
6	4.4±1.4 $\times 10^{-2}$	4.8±1.8	3.1±1.3 $\times 10^{-1}$	2.0±1.4	3.7±2.1

7	4.7±2.1 ×10^{-2}	3.8±1.0	5.2±3.4 ×10^{-1}	0.9±1.0	2.0±1.7
8	1.4±0.3 ×10^{-1}	3.4±0.6	3.0±0.7 ×10^{-1}	2.0±0.7	3.6±0.9
9	1.1±0.1 ×10^{-1}	4.6±0.5	3.2±0.1 ×10^{-1}	2.0±0.6	3.7±0.8
10	1.2±0.3 ×10^{-1}	4.4±1.0	0.3±0.1 ×10^{0}	2.0±1.1	3.7±1.6
11	1.0±0.1 ×10^{-1}	6.2±0.3	4.2±0.7 ×10^{-1}	1.4±0.4	3.0±0.6
12	4.7±0.5 ×10^{-2}	8.9±0.3	4.6±0.6 ×10^{-1}	1.3±0.3	3.0±0.5
13	7.7±0.9 ×10^{-2}	8.1±0.5	3.4±0.5 ×10^{-1}	2.0±1.0	4.6±1.6
14	3.5±0.8 ×10^{-2}	7.4±0.8	3.7±1.1 ×10^{-1}	2.0±1.0	4.6±1.6
15	1.7±0.8 ×10^{-1}	1.6±0.9	3.6±2.2 ×10^{-1}	1.2±1.0	2.0±1.3
16	1.7±0.2 ×10^{-1}	4.3±0.5	2.9±0.5 ×10^{-1}	2.0±0.5	3.6±0.8

Figure S6 includes a compilation of the remaining fitted parameters as a function of the radius of the sphere (a, α, $\bar{k}_1$ and $\bar{k}_2$).

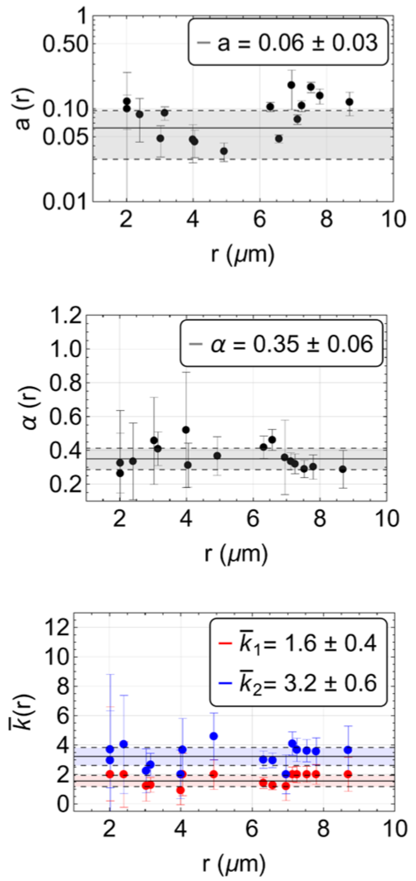

Figure S6. Compilation of parameters a, α, $\bar{k}_1$ and $\bar{k}_2$ (from Table S3) as a function of the radius of the sphere.

Design of the μ-Photoluminescence setup for characterization of coupled devices. An edge detection module was attached to the initial μ-PL setup (Figure S3) to help characterizing SP-waveguide coupled devices. The updated setup is shown in figure S6.

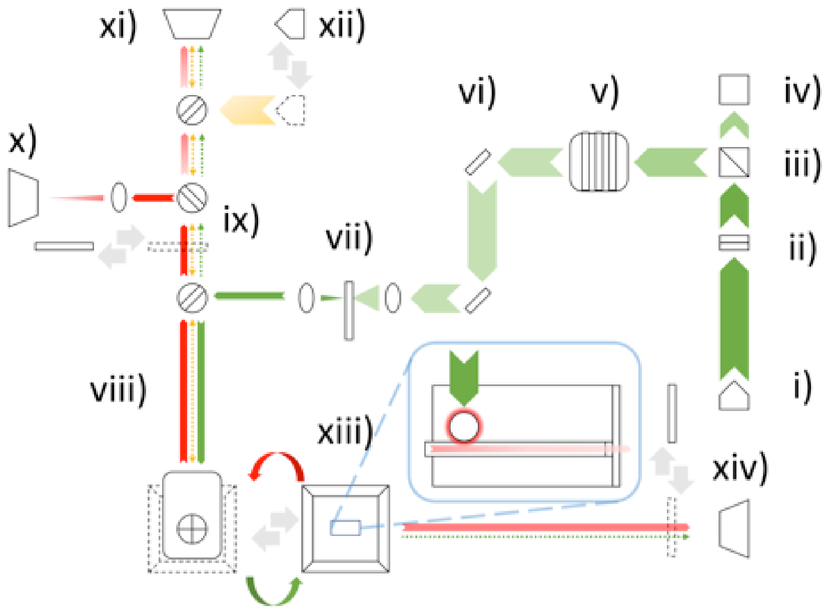

Figure S7. Schematic of the μ-Photoluminescence setup with edge detection: i) Pump source (532 nm laser); ii) Waveplate; iii) Polarised beam splitter; iv) Beam dump; v) set of neutral density filters; vi) mirrors; vii) beam expander with attenuator wheel incorporated; viii) setup with a mounted objective lens (4×/0.13) and a xyz stage where the sample is placed; ix) set of 3 beam splitters and a long pass filter (550 nm); x) Spectrometer

fibre-coupled to the setup; xi) CCD camera; xii) lamp; xiii) detail of SP-waveguide coupling; xiv) CCD camera with a long pass filter incorporated (550 nm). Green arrows represent the path of the laser from the pump, red arrows represent the path of the laser from the SP and yellow arrows represent the light path of the lamp.

This setup has an extra CCD camera mounted on a xyz stage, which is used to focus the image on the end facet of the waveguide to which the SP is coupled to. When the SP is pumped, if emitted light is coupled onto the waveguide, it will travel all the way through and be visible at the end of the facet.

SNR processing

To better visualize the light at the end facet of the waveguide in the main manuscript, the acquired pictures had gamma, contrast and brightness corrections. Figure S8 shows the original pictures that were used for the SNR processing (noise and signal), together with the adjusted pictures (signal with corrections).

Energy pump (nJ)	Noise	Signal	Signal (with corrections)
13.6			
8.5			
5.7			
3.9			

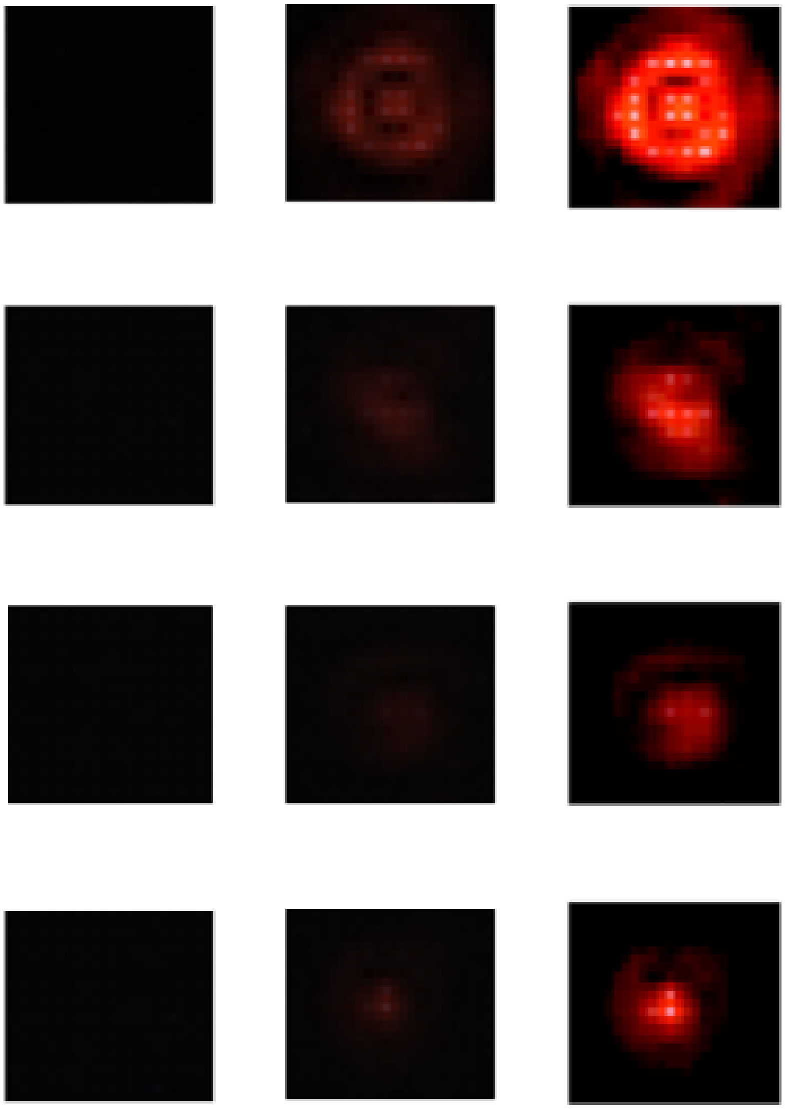

Figure S8. Original pictures of the waveguide facets acquired by the CCD camera and used for the SNR processing calculations (noise and signal). The signal with corrections

used gamma, contrast and brightness adjustments to visualise the coupled modes. SPs were optically pumped at λ_{pump} = 532 nm. The full optical setup can be seen in Figure S7.

Characterization of the transfer printed SP. The fit of the transfer-printed SP (radius of 3.9 ± 0.5 µm) can be seen in Figure S9, together with the fit results and estimated average number of excitons at laser threshold.

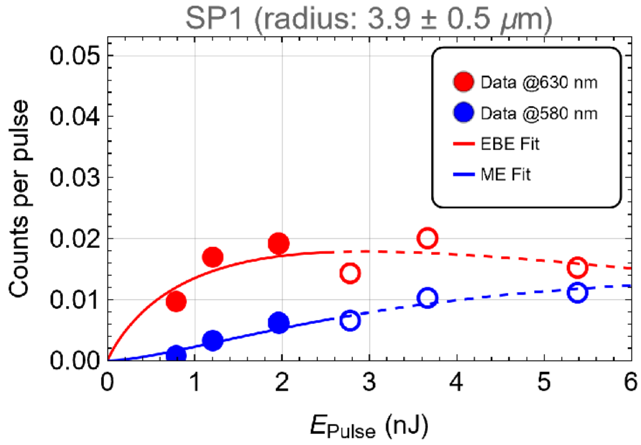

Figure S9. Fit of Eq. 5 to the exciton/biexciton peaks ($\bar{k}_1$) and multiexciton peaks ($\bar{k}_2$) for SPs of different sizes. The circles correspond to the extrapolation data above laser threshold, which match the fits (dashed lines). Fit results: $a = (1.03 \pm 5.25) \times 10^{-3}$,

$\alpha = (0.58 \pm 3.61) \times 10^{-3}$, $b = (9.77 \pm 7.91) \times 10^{-3}$, $\bar{k}_1 = 1.69 \pm 19.2$ and $\bar{k}_2 = 3.21 \pm 28.6$. Using Eq. 2, the estimated average number of excitons in the SP at laser threshold corresponds to $\langle N \rangle = 1.71$.

REFERENCES

(1) Meng, Y.; Zhang, Z.; Yin, H.; Ma, T. Automatic Detection of Particle Size Distribution by Image Analysis Based on Local Adaptive Canny Edge Detection and Modified Circular Hough Transform. Micron 2018, 106 (August 2017), 34–41. https://doi.org/10.1016/j.micron.2017.12.002.

(2) Jevtics, D.; Hurtado, A.; Guilhabert, B.; McPhillimy, J.; Cantarella, G.; Gao, Q.; Tan, H. H.; Jagadish, C.; Strain, M. J.; Dawson, M. D. Integration of Semiconductor Nanowire Lasers with Polymeric Waveguide Devices on a Mechanically Flexible Substrate. Nano Lett 2017, 17 (10), 5990–5994. https://doi.org/10.1021/acs.nanolett.7b02178.

(3) Dement, D. B.; Puri, M.; Ferry, V. E. Determining the Complex Refractive Index of Neat CdSe/CdS Quantum Dot Films. Journal of Physical Chemistry C 2018, 122 (37), 21557–21568. https://doi.org/10.1021/acs.jpcc.8b04522.

(4) Balac, S.; Féron, P. Whispering Gallery Modes Volume Computation in Optical Micro-Spheres; 2014. https://doi.org/hal-01279396v2.
